# Genome‐wide association analysis of spike and kernel traits in the U.S. hard winter wheat

**DOI:** 10.1002/tpg2.20300

**Published:** 2023-01-13

**Authors:** Jyotirmoy Halder, Harsimardeep S. Gill, Jinfeng Zhang, Rami Altameemi, Eric Olson, Brent Turnipseed, Sunish K. Sehgal

**Affiliations:** ^1^ Dep. of Agronomy, Horticulture & Plant Science South Dakota State Univ. Brookings SD 57007 USA; ^2^ Dep. of Plant, Soil and Microbial Sciences Michigan State Univ. East Lansing MI 48824 USA

## Abstract

A better understanding of the genetic control of spike and kernel traits that have higher heritability can help in the development of high‐yielding wheat varieties. Here, we identified the marker‐trait associations (MTAs) for various spike‐ and kernel‐related traits in winter wheat (*Triticum aestivum* L.) through genome‐wide association studies (GWAS). An association mapping panel comprising 297 hard winter wheat accessions from the U.S. Great Plains was evaluated for eight spike‐ and kernel‐related traits in three different environments. A GWAS using 15,590 single‐nucleotide polymorphisms (SNPs) identified a total of 53 MTAs for seven spike‐ and kernel‐related traits, where the highest number of MTAs were identified for spike length (16) followed by the number of spikelets per spike (15) and spikelet density (11). Out of 53 MTAs, 14 were considered to represent stable quantitative trait loci (QTL) as they were identified in multiple environments. Five multi‐trait MTAs were identified for various traits including the number of spikelets per spike (NSPS), spikelet density (SD), kernel width (KW), and kernel area (KA) that could facilitate the pyramiding of yield‐contributing traits. Further, a significant additive effect of accumulated favorable alleles on the phenotype of four spike‐related traits suggested that breeding lines and cultivars with a higher number of favorable alleles could be a valuable resource for breeders to improve yield‐related traits. This study improves the understanding of the genetic basis of yield‐related traits in hard winter wheat and provides reliable molecular markers that will facilitate marker‐assisted selection (MAS) in wheat breeding programs.

## Core Ideas

- Final grain yield in wheat is primarily determined by various kernel and spike traits.
- Fifty‐three MTAs were identified for spike and kernel traits in hard winter wheat germplasm.
- Fourteen MTAs for different traits were consistent over multiple environments.
- Promising germplasm and SNPs associated with yield‐contributing traits will facilitate future wheat breeding.

**Abbreviations**
BLUE: best linear unbiased estimate
CEnv: combined environment
FarmCPU: fixed and random model circulating probability unification
GWAS: genome‐wide association studies
HWWAMP: hard winter wheat association mapping panel
KA: kernel area
KL: kernel length
KW: kernel width
LD: linkage disequilibrium
MAS: marker‐assisted selection
MTA: marker–trait association
NKPS: number of kernels per spike
NSPS: number of spikelets per spike
SD: spikelet density
SL: spike length
SNP: single‐nucleotide polymorphism
TKW: 1,000‐kernel weight

## 1 INTRODUCTION

Bread wheat (*Triticum aestivum* L.) is one of the most important and widely grown food crops, playing a crucial role in feeding 35% of the world's population by providing 19% of calories and 21% of proteins (Tadesse et al., 2019). To meet the growing demand, global food production needs to double by 2050; however, the average annual yield increase of wheat has been reported to be 1%, which is far less than the required rate (1.7%) (Pal et al., 2022; Tadesse et al., 2019). Therefore, to feed an ever‐increasing world population with a gradual decrease in farmland, yield improvement remains the primary focus for wheat breeding programs globally.

Wheat yield is a complex phenomenon influenced by various factors, such as morphological characteristics, physiological indices, grain‐related traits, and different environmental conditions, making yield enhancement increasingly challenging (Liu et al., 2018b; Nadolska‐Orczyk et al., 2017). However, grain yield can be dissected into different component traits such as the number of spikelets per spike (NSPS), spike length (SL), spike number per unit area, number of kernels per spike (NKPS), kernel size, and 1,000‐kernel weight (TKW), which are less sensitive to the environment and have higher heritability than that of grain yield itself (Hai et al., 2008; Kato et al., 2000). Three major components that collectively determine the final yield of wheat are the number of spikes per unit area, NKPS, and TKW (Liu et al., 2018b). In addition, other spike‐related traits, like spike number per plant, SL, and spikelet number, also play a significant role in wheat yield improvement (Guo et al., 2017). Therefore, the identification and further deployment of genomic regions that control different yield‐related traits are essential in dissecting the genetic basis of yield and the overall improvement of wheat.

Wheat yield and yield‐contributing traits exhibit polygenic inheritance and are usually controlled by multiple genes and quantitative trait loci (QTL) (Anuarbek et al., 2020; Jin et al., 2020). Linkage mapping and genome‐wide association studies (GWAS) have been intensively used to dissect the genetic basis of complex traits (Liu et al., 2018a). Further, the effectiveness of GWAS has already been established to capture genetic factors for various traits in wheat including agronomic (Sukumaran et al., 2015; Sun et al., 2017), disease resistance (Arruda et al., 2016; Halder et al., 2019; Juliana et al., 2018; Zhang et al., 2022), and end‐use quality (Chen et al., 2019). Likewise, these approaches have been used to identify significant QTL for several yield‐related traits in wheat (Singh et al., 2022). Quantitative trait loci and genes associated with spike‐ (Cui et al., 2014; Gao et al., 2015; Guo et al., 2017; Huang et al., 2006; Liu et al., 2017b; Naruoka et al., 2011; Pang et al., 2020; Ward et al., 2019) and kernel‐related traits (Chen et al., 2016; Jaiswal et al., 2015; Liu et al., 2018b; Zhang et al., 2012) have been identified on all the wheat chromosomes. Numerous previous studies reported major effect genes and QTL, such as *TaGS5*, *TaSus1*, *TaSus2*, and *TaGW2*, significantly associated with kernel size, kernel weight, spike, peduncle length, and grain weight (Bednarek et al., 2012; Hou et al., 2014; Ma et al., 2016; Wang et al., 2015; Zhang et al., 2018). Two major genes responsible for modern wheat spike morphology (*Q* and *C*) mapped on chromosomes 5A and 2D were also found associated with grain size, shape, grain number, grain yield, and TKW (Johnson et al., 2008; Xie et al., 2018). Genes such as *TaMOC1‐7A*, *TaTEF‐7A*, and *WAPO‐A1* were found to be associated with spikelet or grain number per spike (Kuzay et al., 2019, 2022; Zhang et al., 2015; Zheng et al., 2014), whereas *TaCKX6‐D1* and *TaSAP1* have been reported to have significant effect on TKW and NKPS (Chang et al., 2013; Zhang et al., 2012).

Even though a considerable number of studies dissected the genetics of yield‐related traits in wheat, relatively few studies used winter wheat germplasm (Dhakal et al., 2021; Gill et al., 2022; Lozada et al., 2017; Ward et al., 2019; Zanke et al., 2015). As winter and spring wheat possess considerable genetic differences with respect to growth habits and adaptation, the genetic exchange between these two major wheat classes is limited. The winter wheat breeding programs mostly breed within the winter class and hybridizations among these two classes are made only to introgress specific traits like disease resistance or other marker‐linked traits of agronomic importance. This necessitates the identification of genomic regions related to yield and associated traits within winter wheat for direct use. Further, hard winter wheat germplasm likely harbors many new QTL affecting the yield and contributing traits that are yet to be explored.

**Core Ideas**

- Final grain yield in wheat is primarily determined by various kernel and spike traits.
- Fifty‐three MTAs were identified for spike and kernel traits in hard winter wheat germplasm.
- Fourteen MTAs for different traits were consistent over multiple environments.
- Promising germplasm and SNPs associated with yield‐contributing traits will facilitate future wheat breeding.

The present study used an association mapping panel comprising hard winter wheat accessions from the U.S. Great Plains breeding programs, which has been successfully used to dissect various important traits (Altameemi et al., 2021; Navrotskyi et al., 2020; Sidhu et al., 2020). We sought to perform GWAS using the hard winter wheat association mapping panel (HWWAMP) to identify the genomic regions associated with various spike‐ and kernel‐related traits and explore the putative candidate genes harbored in these regions using the wheat reference genome.

## 2 MATERIALS AND METHODS

### 2.1 Plant materials and field trials

In the current study, we used 297 accessions of the HWWAMP developed under the USDA–TCAP project (Guttieri et al., 2015). This HWWAMP comprises advanced breeding lines and released varieties since the 1940s from the Great Plains region of the United States including Montana, North Dakota, South Dakota, Nebraska, Kansas, Colorado, Oklahoma, and Texas. The association mapping panel was evaluated at three experimental stations of South Dakota State University at the Aurora Farm (E1), Brookings Farm (E2), and Felt Farm (E3) in South Dakota during the 2019–2020 growing season using a randomized complete block design with two replications. Each accession was planted in a 1.25 m long two‐row plot with an interrow spacing of 20 cm. The trials were managed using recommended agronomic practices for proper growth and development.

### 2.2 Phenotypic trait evaluation and statistical analysis

Eight morphological traits were evaluated including NSPS, SL, spikelet density (SD), NKPS, TKW, kernel length (KL), kernel width (KW), and KA. Ten random spikes from each accession per replication were manually harvested at physiological maturity. Spike length was measured from the base of the rachis to the topmost spikelet excluding the awns. The NSPS were counted from the basal sterile spikelet to the top fertile spikelet. The SL and NSPS were the means of 10 randomly selected spikes in each replication. Spikelet density was calculated as the ratio of NSPS to SL. The TKW was measured by weighing 1,000 kernels from each accession in two replicates. Three kernel‐related traits (KL, KW, and KA) were recorded following an automatic grain analyzer Vibe QM3 (Vibe Imaging Analytics).

META‐R (Multi Environment Trial Analysis with R) v6.04 (Alvarado et al., 2020) was used for estimating the best linear unbiased estimates (BLUEs) for all the traits. The BLUEs of two replicates for individual locations were estimated using the following model:

$$\;y_{ij}=\mu +G_{i}+R_{j}+e_{ij}$$

where *y_ij_* is the trait of interest, μ is the overall mean, *G_i_* is the fixed effect of the *i*th genotype or accession, *R_j_* is the random effect of the *j*th replicate, and *e_ij_* is the random error term associated with the *i*th genotype and *j*th replicate. For multilocation analysis, BLUEs over three environments were obtained using the following model:

$$\;y_{ijk}=\mu +G_{i}+E_{j}+R_{k\left(j\right)}+{\mathrm{GE}}_{ij}+e_{ijk}$$

where *y_ijk_* is the trait of interest, μ is the overall mean, *G_i_* is the fixed effect of the *i*th genotype, *E_j_* is the random effect of the *j*th environment, *R _k_*_(_ *_j_*_)_ is the random effect of the *k*th replicate nested within the *j*th environment, GE*_ij_* is the random effect of the genotype × environment (G × E) interaction, and *e_ijk_* is the random error term. To estimate the broad‐sense heritability (*H* ^2^) across environments, variance components were estimated by fitting genotype (*G_i_*) effects as random. The heritability was calculated as follows:

$$H^{2}=\frac{{\hat{\sigma }}_{g}^{2}}{{\hat{\sigma }}_{g}^{2}+{\hat{\sigma }}_{\mathrm{gE}}^{2}/n\mathrm{Loc}+{\hat{\sigma }}_{e}^{2}/\left(n\mathrm{Loc}\times n\mathrm{Rep}\right)}$$

where ${\hat{\sigma }}_{g}^{2}$ genotype variance component, and ${\hat{\sigma }}_{\mathrm{gE}}^{2}$ is G × E interaction variance component, and ${\hat{\sigma }}_{e}^{2}$ is the error variance components, respectively. The *n*Rep term represents the number of replications within an environment and *n*Loc term represents the number of environments in the analysis.

### 2.3 Genotyping and SNP discovery

The HWWAMP was genotyped using the wheat Infinium 90K iSelect array (Illumina Inc.) under the USDA–TCAP (Cavanagh et al., 2013). The genotypic data was obtained from the T3 Toolbox (https://wheat.triticeaetoolbox.org/). The dataset was then filtered to remove the single‐nucleotide polymorphisms (SNPs) with any heterozygosity, >10% missing data, and minor allele frequency of <5%. The filtered genotypic dataset consisted of a total of 15,590 high‐quality SNPs, which were then imputed in TASSEL v5.0 (Bradbury et al., 2007) for further analysis. The genetic positions of the wheat Infinium 90K iSelect SNP markers were obtained from the consensus genetic map of 46,977 SNPs (Wang et al., 2014). The physical positions of the SNPs associated with various spike‐ and kernel‐related traits were obtained by BLAST searching the flanking sequences of respective SNPs to wheat Chinese Spring RefSeq v1.1 (IWGSC et al., 2018).

### 2.4 Population structure and linkage disequilibrium

Population structure among the 297 hard winter wheat accessions was assessed using a Bayesian model‐based clustering program STRUCTURE v2.3.4 assuming an admixture model (Pritchard et al., 2000). The most likely number of subgroups was inferred based on an ad hoc statistic (Delta*K*) based on the rate of change in the log probability between runs using successive *K* values (Evanno et al., 2005) using STRUCTURE HARVESTER (Earl & vonHoldt, 2012). We used 10 subgroups (*K* = 1–10) with five independent runs for each subgroup using a burn‐in period of 10,000 iterations followed by 10,000 Monte Carlo iterations. The HWWAMP was analyzed for linkage disequilibrium (LD) decay pattern in one of our previous studies (Ayana et al., 2018) using a set of 1,842 SNPs in TASSEL v5.0 (Bradbury et al., 2007). The LD decay was estimated as the distance where the LD value (*r* ^2^) falls below 0.1 or half strength of D' (D' = 0.5). For the whole genome, the LD dropped to 0.5 at 4.5 cM. The LD decay was found similar for both A (3.4 cM) and B (3.6 cM) subgenomes; however, it was much higher for the D subgenome (14.2 cM).

### 2.5 Marker–trait associations

Genome‐wide associations were analyzed using two different algorithms, namely the mixed linear model (Yu et al., 2006) and fixed and random model circulating probability unification (FarmCPU) (Liu et al., 2016). Both the models were implemented in the Genomic Association and Prediction Integrated Tool (GAPIT) (Lipka et al., 2012). In brief, the mixed linear model incorporates kinship and population structure as covariates to minimize the confounding effects and control false positives. However, it leads to false negatives because of the confounding between testing markers and cofactors simultaneously. The FarmCPU model is an improved multiple‐locus model that controls false positives by fitting the associated markers detected from the iterations as cofactors to perform marker tests within a fixed‐effect model. The quantile–quantile plots suggested that the FarmCPU performed better than the mixed linear model for most traits. Therefore, we employed FarmCPU to report the marker–trait associations (MTAs) for all the spike‐ and kernel‐related traits. Though we found several associations to be significant based on the Bonferroni correction for multiple testing, most of these associations were limited to one or two environments. Thus, we used an arbitrary threshold of −log_10_ *P* ≥ 3.0 to report MTAs following previous studies (Dong et al., 2016; Liu et al., 2017a; Sidhu et al., 2020; Wang et al., 2017), and only those MTAs were reported that surpassed this threshold in the combined environment analysis (CEnv) and at least two individual environments.

Further, we sought to analyze the effect of accumulated favorable alleles on the trait's phenotype. The accessions from the mapping panel were grouped based on the alleles of identified MTAs for all the spike‐related traits. These groups were compared using Tukey's HSD (honestly significant difference) test at a 5% level of significance to verify the additive effect of the favorable alleles on the phenotype of the traits.

### 2.6 Identification of candidate genes

For candidate gene analysis, only stable MTAs for three spike traits were selected. The candidate regions were demarcated within ±1 Mb of the most significant SNP for each region to identify the candidate genes. The high‐confidence genes in the selected region were retrieved from the wheat genome assembly IWGSC RefSeq v1.1 and gene ontology annotation information of these genes was extracted from IWGSC Functional Annotation v1.0 (IWGSC et al., 2018). The gene expression browser was used to exclude unlikely candidates and shorten the candidate list to fewer genes for each selected MTA (Ramírez‐González et al., 2018).

## 3 RESULTS

### 3.1 Phenotypic variation and correlations

**FIGURE 1:**
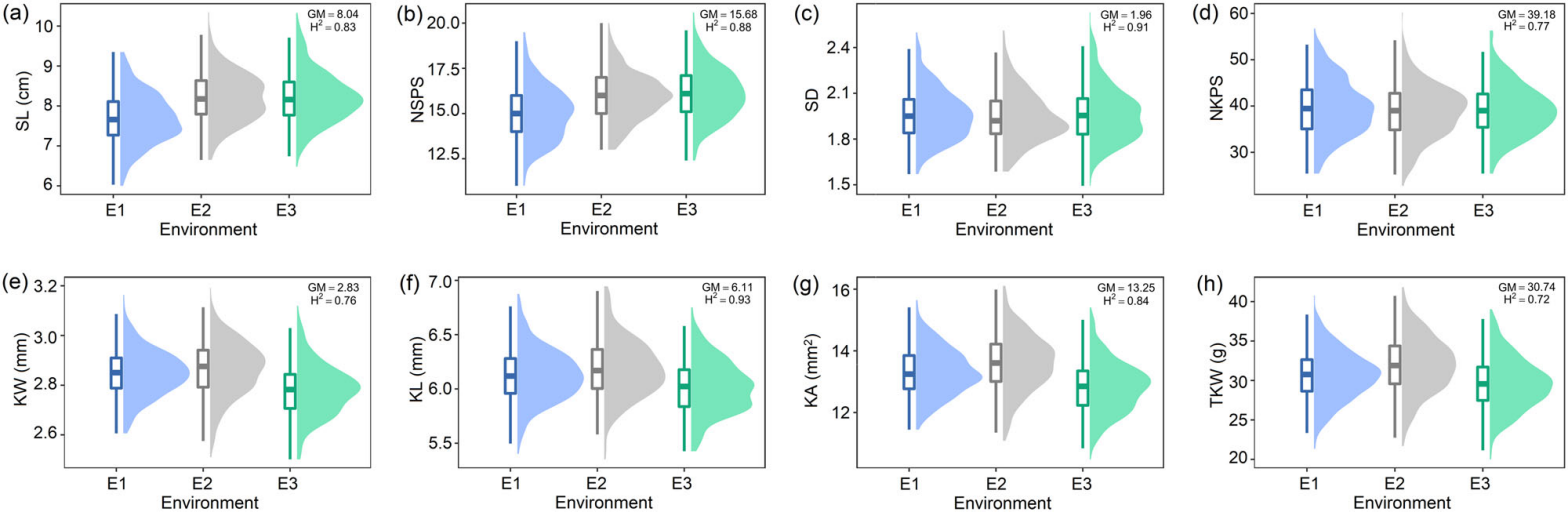
Distributions of eight spike‐ and kernel‐related traits in three different environments (E1, E2, and E3). (a) spike length (SL); (b) number of spikelets per spike (NSPS); (c) spikelet density (SD); (d) number of kernels per spike (NKPS); (e) kernel width (KW); (f) kernel length (KL); (g) kernel area (KA); and (h) thousand‐kernel weight (TKW). GM, grand mean across three environments; *H* ^2^, broad‐sense heritability

**FIGURE 2:**
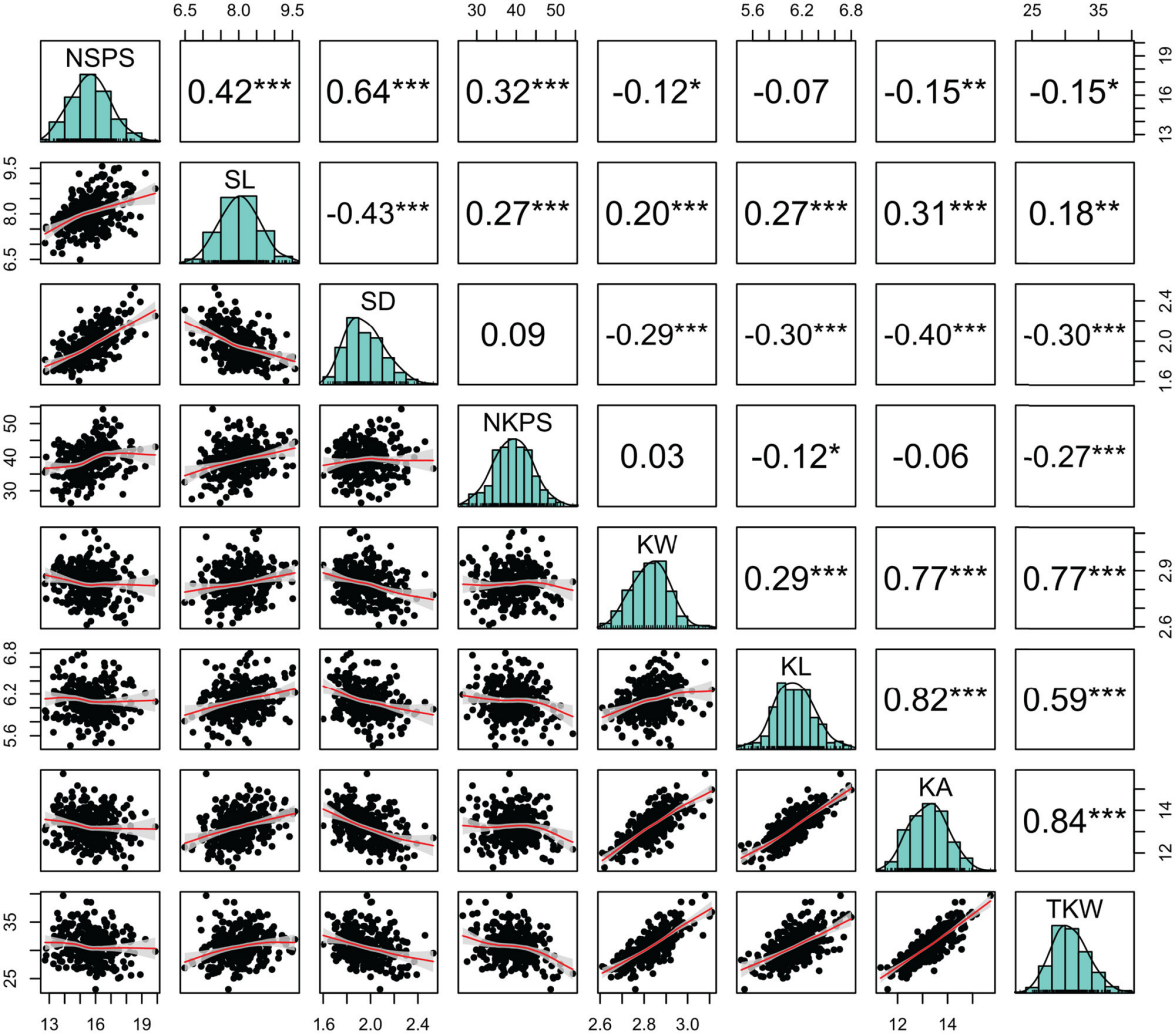
Pearson's correlation matrix among spike‐ and kernel‐related traits based on their best linear unbiased estimates. Values inside the rectangle represent the correlation coefficient and three symbols *, **, and ***, represent correlation coefficient significance levels at α <.05, .01, and .001, respectively. Bivariate scatter plots with fitted lines are on the left side of the diagonally arranged phenotypic distribution plots. NSPS, number of spikelets per spike; SL, spike length; SD, spikelet density; NKPS, number of kernels per spike; KW, kernel width; KL, kernel length; KA, kernel area; TKW, thousand‐kernel weight

A wide variation was observed in the HWWAMP for all the spike‐ and kernel‐related traits across three environments (Supplemental Table S1). Overall, phenotypic variabilities of traits appeared to be normally distributed with noticeable variation among three environments (Figure 1). Correlation coefficients among three different environments and CEnv values for each trait were generally high (Supplemental Figures S1 and S2). The broad‐sense heritability estimates for eight traits ranged from .72 (TKW) to .93 (KL). Pearson's correlation among eight traits was estimated using across‐environment BLUE values (Figure 2). A moderate to high positive correlation was observed between NSPS and other spike‐related traits such as SL (*r* = 0.42), SD (*r* = 0.64), and NKPS (*r* = 0.32). However, NSPS was negatively correlated with all four kernel‐related traits (KW, KL, KA, and TKW). On the other hand, SL showed significant positive correlations with all the studied traits except SD (*r* = −0.43) (Figure 2). As expected, we observed a strong positive correlation within four kernel‐related traits (KW, KL, KA, and TKW), and kernel‐related traits showed a negative correlation with spike‐related traits except for SL (Figure 2).

### 3.2 Genotyping and population structure

The genotype data was filtered to obtain a total of 15,590 high‐quality SNPs that were distributed across all 21 wheat chromosomes (Supplemental Table S2). We investigated the population structure to assess if the association‐mapping panel is structured based on the breeding programs and origin and to figure out any relationship of population structure with the spike and kernel traits. Four subpopulations (P1, P2, P3, and P4) were identified in the HWWAMP, where P1, P2, P3, and P4 consist of 120, 34, 33, and 110 genotypes. We found a significant difference in means among the four subpopulations (P1, P2, P3, and P4) for all the traits except SL (Supplemental Table S3).

### 3.3 GWAS on the spike‐ and kernel‐related traits

A GWAS was performed on the spike‐ and kernel‐related traits using BLUEs from across‐environment analysis (CEnv) and individual environments (Supplemental Figure S3).

**FIGURE 3:**
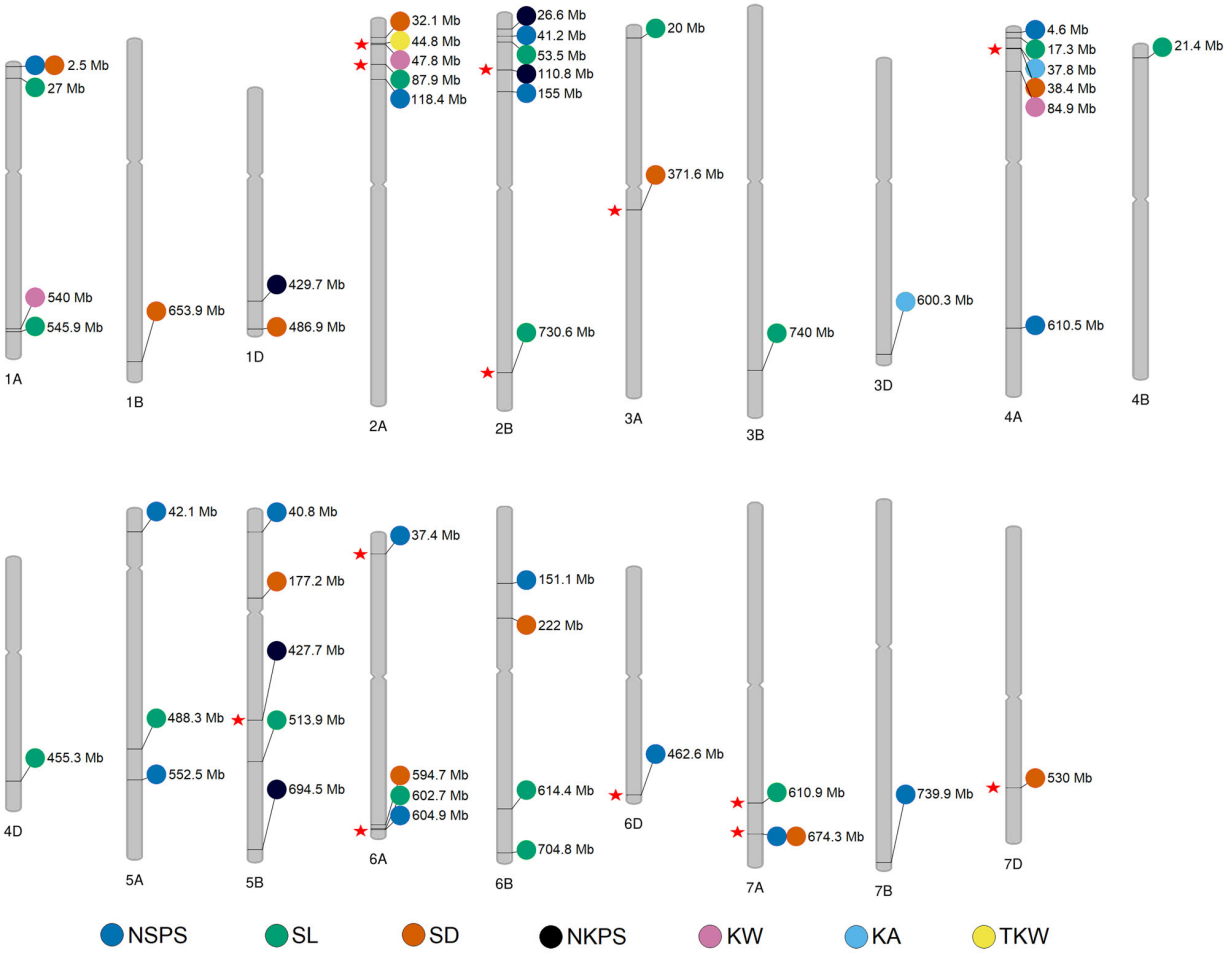
Physical position (Mb) of the marker–trait associations (MTAs) identified for seven spike‐ and kernel‐related traits in the combined analysis (CEnv). The physical positions are based on Chinese Spring RefSeq 1.1 (IWGSC et al., 2018). The consistent MTAs for respective traits identified over multiple environments are highlighted using a red star on left side of the chromosome bars. NSPS, number of spikelets per spike; SL, spike length; SD, spikelet density; NKPS, number of kernels per spike; KW, kernel width; KA, kernel area; TKW, thousand‐kernel weight

The GWAS identified a total of 53 MTAs (*P* < .001) in combined environment analysis (CEnv) for seven traits out of eight studied traits, as no MTA was observed for KL (Supplemental Table S4). The MTAs were distributed across all the chromosomes except 2D and 5D (Figure 3). Most of the MTAs were identified on the A subgenome (28), followed by the B (19) and D (6) subgenomes. The highest number of MTAs were located on chromosomes 2B and 4A (six MTAs each), followed by 1A, 2A, and 3B (five MTAs each) (Figure 3; Supplemental Table S4). Of the 53 MTAs from the CEnv analysis, we emphasized only on 14 MTAs that were identified in CEnv as well as at least two individual environments (Table 1). Thus, these 14 MTAs were considered to represent stable QTL for spike or kernel traits in U.S. hard winter wheat breeding germplasm, whereas the 39 MTA identified in CEnv could be considered putative QTL (Supplemental Table S4).

**TABLE 1:** Stable marker–trait associations identified in multiple environments (CEnv and at least two individual environments) for various spike‐ and kernel‐related traits in the U.S. hard winter wheat

Trait	SNP^a^	Chr	Alleles^b^	Pos^c^	−Log_10_ *P*	Environments^d^	Effect
NSPS	*Excalibur_c97022_396*	6A	C/T	37,415,157	4.0	E1, E3, CEnv	−0.266
	*RFL_Contig3175_1217*	6A	T/C	604,877,158	3.5	E2, E3, CEnv	−0.168
	*IWA4455*	6D	A/G	462,631,946	3.8	E1, E3, CEnv	0.061
	*IWA5913*	7A	A/G	674,276,906	14.0	E1, E2, E3, CEnv	−0.500
SL	*Kukri_c10860_1283*	2A	G/A	87,857,405	4.7	E1, E2, CEnv	0.163
	*Tdurum_contig82393_484*	2B	C/A	730,562,664	8.4	E1, E3, CEnv	0.147
	*IWA3639*	7A	G/A	610,934,198	7.6	E1, E3, CEnv	−0.138
SD	*IWA2519*	3A	C/T	371,628,644	3.3	E1, E2, CEnv	−0.026
	*IWA5913*	7A	A/G	674,276,906	9.4	E1, E2, E3, CEnv	−0.055
	*IWA1902*	7D	A/G	530,035,575	4.1	E1, E2, E3, CEnv	−0.041
NKPS	*BS00021959_51*	2B	C/T	110,818,850	3.2	E1, E3, CEnv	−1.862
	*Excalibur_c1921_1191*	5B	G/A	427,650,909	4.0	E1, E2, CEnv	1.390
KW	*BS00044274_51*	2A	T/G	47,826,702	3.3	E2, E3, CEnv	0.024
KA	*Kukri_c74409_199*	4A	G/A	37,773,890	3.4	E1, E3, CEnv	0.250

*Note*. Chr, chromosome; Pos, physical position in base pair (based on IWGSC RefSeq); NSPS, number of spikelets per spike; SL, spike length; SD, spikelet density; NKPS, number of kernels per spike; KW, kernel width; KA, kernel area.

^a^ Single‐nucleotide polymorphisms (SNPs) were significant in multiple environments.

^b^ Favorable allele is underlined.

^c^ Position in base pair (bp) based on Chinese Spring RefSeq 1.1.

^d^ Environment 1 (E1), Environment 2 (E2), Environment 3 (E3), combined best linear unbiased estimates (CEnv).

#### 3.3.1 Spike‐related traits

Of the 15 MTAs identified for NSPS in CEnv (Supplemental Table S4), four MTAs, namely *Excalibur_c97022_396* (37.4 Mb), *RFL_Contig3175_1217* (604.9 Mb), *IWA4455* (462.6 Mb), and *IWA5913* (674.3 Mb) on chromosomes 6A, 6A, 6D, and 7A, respectively, were identified in multiple environments (Table 1). For SL, a total of 16 MTAs (Supplemental Table S4) were identified of which only three SNPs, namely *Kukri_c10860_1283* (87.9 Mb), *Tdurum_contig82393_484* (730.6 Mb), and *IWA3639* (610.9 Mb) on chromosomes 2A, 2B, and 7A, were identified in multiple environments (Table1). Out of the 11 MTAs for SD in CEnv analysis, three MTAs, including *IWA2519* (371.6 Mb), *IWA5913* (674.3 Mb), and *IWA1902* (530 Mb) on chromosomes 3A, 7A, and 7D, were identified in multiple environments. Similarly, we identified five MTAs for NKPS on chromosomes 1D, 2B, and 5B, of which *BS00021959_51* (110.8 Mb) and *Excalibur_c1921_1191* (427.7 Mb) on chromosomes 2B and 5B were identified in multiple environments (Supplemental Table S4; Figure 3).

#### 3.3.2 Kernel‐related traits

The GWAS identified only six MTAs for three (KW, KA, and TKW) kernel‐related traits (Supplemental Table S4, Supplemental Figure S3). In total, three MTAs were detected for KW on chromosomes 1A, 2A, and 4A. Out of the three MTAs, only *BS00044274_51* (47.8 Mb) on chromosome 2A was significant in CEnv and at least two environments (Table 1). Further, two MTAs were identified for KA on chromosomes 3D and 4A; however, only *Kukri_c74409_199* (37.8 Mb) on chromosome 4A was found in multiple environments and CEnv. We identified only one MTA for TKW located on chromosome 2A (Supplemental Table S4).

### 3.4 Genomic regions or SNPs associated with multiple traits

We identified a total of five MTAs that were associated with more than one trait (Supplemental Table S4). Two MTAs, namely *Excalibur_c35316_154* (2.5 Mb) and *IWA5913* (674.2 Mb) located on chromosomes 1A and 7A, were associated with both NSPS and SD. Similarly, *BS00044274_51* (47.8 Mb) on chromosome 2A was associated with KA and KW, whereas *IWA6659* (84.9 Mb) on chromosome 4A was associated with several spike‐ and kernel‐related traits including KW, KA, TKW, and SL. Another MTA, *IWA6485* (600.2 Mb) on chromosome 3D, was significantly associated with KA and SL (Supplemental Table S4).

### 3.5 Phenotypic effects of favorable alleles of detected MTAs

**FIGURE 4:**
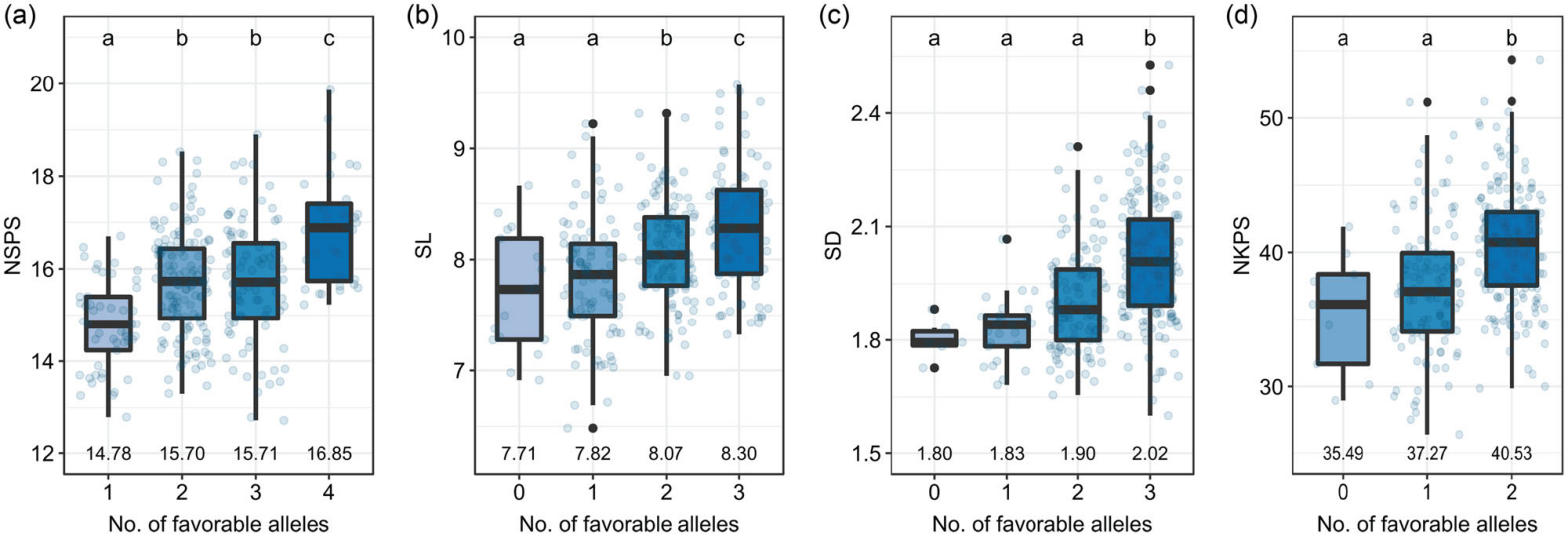
Effect of accumulation of favorable alleles of the identified stable associations (marker–trait associations identified in multiple environments) for four spike‐related traits: (a) number of spikelets per spike (count); (b) spike length (cm); (c) spikelet density; and (d) kernel per spike (count). The *x* axis represents the favorable allele count and *y* axis represents the respective phenotypic value. Significantly different groups are represented with different letters (on top of the boxplots) based on Tukey's HSD test at .05 level of significance. NSPS, number of spikelets per spike; SL, spike length; SD, spikelet density; NKPS, number of kernels per spike

To identify the accumulative effect of favorable alleles for various traits, we grouped the HWWAMP based on the number of favorable alleles carried by the accessions for four spike‐related traits (Figure 4). We used only stable MTAs identified for spike‐related traits in this analysis (Table 1). For NSPS, accessions with one, two, three, and four favorable alleles had a mean value of 14.78, 15.70, 15.71, and 16.85 spikelets per spike, respectively. The accessions with three or more favorable alleles had significantly higher NSPS than lines with only one favorable allele (Figure 4). We also observed a significant difference in SL with the increment of favorable alleles. The HWWAMP lines that carried none of the identified favorable alleles for SL had a mean of 7.7 cm, whereas lines with one, two, and three favorable alleles had a mean SL of 7.82, 8.06, and 8.30 cm, respectively. A similar additive effect was observed for SD, as three favorable alleles significantly increased the compactness of the spike (SD) compared with lines with a lower number of favorable alleles. Similarly, the trait mean for NKPS was 35.49, 37.27, and 40.53 when the accessions had zero, one, and two favorable alleles, respectively (Figure 4).

### 3.6 Candidate gene analysis for stable MTAs

Candidate gene analysis was performed for five stable MTAs having the −log_10_ *P* ≥ 4.0. For each MTA, a window of ±1 Mb around the most significant SNP was demarcated to identify the candidate genes. A total of 120 high‐confidence genes were retrieved by using IWGSC RefSeq v1.0 annotation. However, we excluded the unlikely candidates using publicly available RNA sequencing expression data from the Wheat Expression Browser and a thorough review of related literature. Finally, 14 putative candidate genes were selected for three different traits based on their relatively high expression in the shoot, spike, and grain development at the vegetative and reproductive stages of wheat (Table 2). The selected genes encode for various proteins including peptidyl‐prolyl cis‐trans isomerase, cold shock protein, NADPH‐cytochrome P450 reductase, glycoprotein membrane GPI‐anchored, ubiquitin‐like protein, BZIP transcription factor, and ATP‐dependent RNA helicase (Table 2).

**TABLE 2:** Putative candidate genes within the identified regions associated with wheat spike‐related traits

Trait	Significant SNP	Chr	Gene ID	Protein
NSPS	*Excalibur_c97022_396*	6A	*TraesCS6A01G068300*	Gamma‐glutamylcyclotransferase
			*TraesCS6A01G068900*	Peptidyl‐prolyl cis‐trans isomerase
			*TraesCS6A01G069500*	Cold shock protein
SL	*Tdurum_contig82393_484*	2B	*TraesCS2B01G533500*	Sulfhydryl oxidase 1
			*TraesCS2B01G534200*	NADPH–cytochrome P450 reductase
			*TraesCS2B01G534700*	UPF0136 membrane protein
	*Kukri_c10860_1283*	2A	*TraesCS2A01G141800*	Transcription‐associated protein 1
			*TraesCS2A01G141900*	Glycoprotein membrane GPI‐anchored (TaMs1)
			*TraesCS2A01G142700*	Ubiquitin‐like protein
			*TraesCS2A01G142800*	BZIP transcription factor
	*IWA3639*	7A	*TraesCS7A01G419100*	60S acidic ribosomal protein P3
			*TraesCS7A01G419400*	ATP‐dependent RNA helicase
SD	*IWA1902*	7D	*TraesCS7D01G411600*	60S acidic ribosomal protein P3
			*TraesCS7D01G412200*	ATP‐dependent RNA helicase

*Note*. NSPS, number spikelets of per spike; SL, spike length; SD, spikelet density, Chr, chromosome.

## 4 DISCUSSION

Wheat yield is the most important and complex target trait for wheat breeding programs. Grain yield mainly depends on the accumulative effect of the different yield‐contributing traits including spike‐ and kernel‐related traits. Breeders have relied upon the identification and deployment of novel genes and QTL governing these crucial traits. Nevertheless, relatively few studies (Dhakal et al., 2021; Lozada et al., 2017; Ward et al., 2019; Zanke et al., 2015) have been reported on winter wheat. In this study, a diverse panel of varieties and advanced breeding lines obtained from various hard winter wheat breeding programs from the U.S. Great Plains was used to dissect the genetics of spike and kernel traits.

### 4.1 Phenotypic correlations among traits and heritability

A wide range of phenotypic variation was observed among the germplasm for all the traits studied, making this panel suitable for GWAS (Supplemental Table S1). High broad‐sense heritability was observed for all the traits ranging from .72 for TKW to .93 for KL, indicating less environmental influence on the observed traits (Figure 1). The high heritability estimates were in line with several previous studies (Alqudah et al., 2020; Garcia et al., 2019; Muhammad et al., 2020; Wang et al., 2017). We observed a significant positive correlation between NSPS and other spike‐related traits (SL, SD, and NKPS), whereas a strong negative correlation was observed between SL and SD (Figure 2). In this study, we observed a moderately positive correlation (0.32) between NSPS and NKPS, which was lower than expected. All of our experiments were conducted in dryland (without irrigation) to mimic the South Dakota and U.S. Great Plains environments. Two of the three environments were drier during flowering and grain‐filling stages likely resulting in several spikelets being sterile, which is in line with previous studies that showed abiotic environmental factors such as drought impairs wheat growth and leads to spikelet sterility (Gol et al., 2021; Ji et al., 2010). In our study, we counted the total number of spikelets (both fertile and sterile). Thus, the lower‐than‐expected correlation between NSPS and NKPS observed in this study might be related to the increased number of sterile spikelets. Spikelet density showed a strong positive relation with NSPS but was negatively correlated with SL, owing to its mathematical dependency on these traits. These results also indicate that the loci controlling these three traits (NSPS, SL, and SD) are often not independent and could be in an inverse relationship. Therefore, to accelerate the breeding process, it is essential to determine relationships among different interdependent traits. On the other hand, all the kernel‐related traits were positively correlated among themselves and with SL, showing that increased SL positively affects kernel traits (Figure 2). Several previous studies also reported positive associations among TKW, KL, and KW (Breseghello & Sorrells, 2007; Chen et al., 2016; Muhammad et al., 2020; Ramya et al., 2010; Rasheed et al., 2014). We also observed that an increase in NSPS, NKPS, and SD causes a decrease in kernel‐related traits (KW, KL, KA, and TKW), which is consistent with the previous reports (Muhammad et al., 2020; Qu et al., 2021; You et al., 2021). Further, two yield‐component traits, grain number and grain weight or KA, are known to be negatively correlated (Bustos et al., 2013; García et al., 2013; Sadras, 2007), and this might be a consequence of trade‐offs between these traits. Further, the competition for assimilates between spikelets leads to an unbalanced distribution of grain number along with the spike (Guo et al., 2017; Molero et al., 2019).

### 4.2 Marker–trait associations and comparison with previous studies

We used the FarmCPU algorithm to identify the MTAs that use both fixed and random effect models iteratively to control the false positives arising from population structure and familial relatedness. In this study, a total of 53 MTAs were identified for the spike‐ and kernel‐related traits using combined BLUEs across three environments (CEnv) (Supplemental Table S4). However, we mainly focused on the MTAs identified in multiple environments and MTAs associated with multiple traits (Table 1). We identified 47 MTAs for spike‐related traits (Supplemental Table S4), while only a few associations were detected for kernel‐related traits. Several MTAs identified in this study were likely to be novel, whereas many of the MTAs colocalized with QTLs that have been reported to be reliable in various previous studies. Spike‐related traits are generally complex and most of the wheat chromosomes harbor genetic factors affecting these traits (Liu et al., 2018b). Therefore, optimization of wheat inflorescence traits, such as head length or SL, spikelet number, and SD can effectively enhance the integrated sink capacity and ultimate yield potential of wheat (Fan et al., 2019; Yao et al., 2019; You et al., 2021).

The MTAs for NSPS were distributed on the chromosomes 1A, 2A, 2B, 4A, 5A, 5B, 6A, 6B, 6D, 7A, and 7B (Supplemental Table S4). Similar to our findings, many previous studies reported several important chromosomes, such as 1A, 5A, 7A, and 7B, harbor significant stable QTL for NSPS (Cui et al., 2012; Hu et al., 2020; Kato et al., 2000; Ma et al., 2007; Liu et al., 2018a). In our study, four stable MTAs, namely *Excalibur_c97022_396* (37.4 Mb), *RFL_Contig3175_1217* (604.9 Mb), *IWA4455* (462.6 Mb), and *IWA5913* (674.3 Mb) on chromosomes 6AS, 6AL, 6DL, and 7AL, respectively (Table 1), were found to be significantly associated with NSPS. Of the four stable associations for NSPS, one MTA (*IWA5913*) identified on chromosome 7A overlapped with *WHEAT ORTHOLOG OF APO1* (*WAPO1*) that was cloned from the wheat chromosome arm 7AL (∼674 Mb) in a recent study as a gene regulating NSPS (Kuzay et al., 2019, 2022). Further, Sun et al. (2017) identified an SNP (*Kukri_c264_539*) for NSPS on 6A (29.5 Mb) in four environments, which is a similar region as the stable MTA *Excalibur_c97022_396* (37.4 Mb) detected in this study. We did not find any QTL reported for NSPS in regions *RFL_Contig3175_1217* (6A) and *IWA4455* (6D), therefore these two stable MTA could be novel. In addition to these stable MTAs, we also identified two other MTAs for NSPS that were only identified in CEnv analysis. One MTA on chromosome 7B (*GENE‐4848_95*; 740 Mb) was colocated in a similar region with a stable QTL reported by Liu et al. (2018a). Moreover, one significant marker (*BS00011235_51*; 552 Mb) for NSPS was detected on chromosome 5A, which is located ∼35 Mb away from the region containing an important gene controlling vernalization (*Vrn‐A1*) (Yan et al., 2003). This *Vrn‐A1* gene has been previously reported to be involved in increasing the NSPS (Whitechurch & Snape, 2003).

In our study, 16 MTAs were identified on chromosomes 1A, 2A, 2B, 3A, 3B, 4A, 4B, 4D, 5A, 5B, 6A, 6B, and 7A for SL. Out of those MTAs, three associations, namely *Kukri_c10860_1283* (87.9 Mb), *Tdurum_contig82393_484* (730.6 Mb), and *IWA3639* (610.9 Mb) on chromosomes 2A, 2B, and 7A, were found to be stable over multiple environments (Table 1). However, MTAs *Kukri_c10860_1283* and *Tdurum_contig82393_484* for SL have been previously reported in various studies (Liu et al., 2018a, 2017b; Mwadzingeni et al., 2017), but we did not find any previous reports on SL QTL in proximity to *IWA3639* (610.9 Mb) on chromosome 7A. Another MTA (*Excalibur_rep_c68588_1196*) identified in the CEnv analysis on chromosome 4BS (21 Mb) for SL, corresponds to the physical location of the semidwarfing gene *Rht‐B1* (∼30 Mb). Other than reducing plant height, *Rht‐B1* was reported to exhibit pleiotropic effects on grain yield and yield components including SL (Guan et al., 2020; Okada et al., 2019). Several other MTAs identified on chromosomes 5A, 6A, and 6B for SL in the CEnv analysis in this study were found to be colocalized with MTAs reported by previous studies (Liu et al., 2018a; Mwadzingeni et al., 2017; Yu et al., 2014) (Supplemental Table S4).

For SD, 11 MTAs distributed on chromosomes 1A, 1B, 1D, 2A, 3A, 4A, 5B, 6A, 6B, 7A, and 7D were identified; however, MTAs on chromosomes 3A, 7A, and 7D were stable and significant in multiple environments (Table 1; Supplemental Table S4). Similar to our finding, several previous studies reported MTAs for SD on chromosomes 1A, 1B, 3A, 4A, 6A, and 6B (Jantasuriyarat et al., 2004; Liu et al., 2018b). The QTL identified for SD, such as *qSc‐7A* (Fan et al., 2019) and *QSd.sau‐2SY‐7A.2* (You et al., 2021), were found in the vicinity of stable association (*IWA5913*) detected in this study on chromosome 7A (674 Mb), the region harboring major NSPS‐associated gene *WAPO1*. Another association for SD (*IWA1902*) at 530 Mb on chromosome 7D was consistently identified in all three individual environments and the CEnv analysis, indicating the importance of this region in spike morphology. A minor QTL for SD on chromosome 7D was also identified in a multiple‐environment joint analysis by Liu et al. (2020) in a similar genomic region as detected in our study. The chromosome 7D MTA could be further studied and validated in different genetic backgrounds and multiple environments to facilitate its application in wheat breeding.

For NKPS, we observed five MTAs that were distributed on chromosomes 1D, 2B, and 5B (Supplemental Table S4). One stable association on chromosome 2B (*BS00021959_51*) was detected at 110.8 Mb (90.9 cM) for NKPS (Table 1). Shi et al. (2017) also reported an association for NKPS at 122.3 Mb and suggested that this marker may be linked to the photoperiod‐insensitive gene *Ppd‐B1* in common wheat. A similar association was detected by Gao et al. (2015) at 92 cM for the same trait on chromosome 2B in a biparental mapping population in two environments. It would be interesting to know if this QTL for NKPS is *Ppd‐B1*. Further, we identified another stable MTA (*Excalibur_c1921_1191*) for NKPS on chromosome 5B at 427.6 Mb (Table 1). Tang et al. (2011) mapped three QTL for NKPS spanned by markers *Xgwm499* and *Xgwm213* (418.8–477.5 Mb) overlapping the physical position identified in our study. Two other MTAs were identified using the CEnv analysis for NKPS on chromosomes 2B (*CAP8_c5108_139*) and 5B (*IWA4329*), which colocalized with the previously reported MTAs in several studies (Guo et al., 2017; Liu et al., 2018a; Neumann et al., 2011) (Supplemental Table S4).

A total of three MTAs were identified for KW on chromosomes 1A, 2A, and 4A. Several studies have reported associations in similar physical locations on chromosomes 1A and 2A for KW (Li et al., 2019; Wu et al., 2015). Further, we also identified MTAs for KA on chromosomes 3D and 4A and for TKW on 2A; however, previous studies also identified significant association for KA and TKW on chromosomes 4A and 2A, respectively (Liu et al., 2018a; Su et al., 2018) (Table 1; Supplemental Table S4). Thus, the present study also validated many of the QTL reported in previous studies from different backgrounds in addition to the identification of several novel QTL for spike and kernel traits.

### 4.3 Multi‐trait QTL regions

We identified several genomic regions that were associated with more than one trait and this phenomenon could likely be due to the complex relationships among these traits (Figure 3). Our study identified two MTAs (*Excalibur_c35316_154* and *IWA5913*) responsible for NSPS, which were also found to be associated with the SD (Supplemental Table S4). In our study, these could be likely a result of the mathematical dependency of SD on NSPS, as SD is expressed as the ratio of NSPS to SL. However several previous studies also reported numerous QTL clusters simultaneously affecting various spike‐related traits and involving major genes (Cui et al., 2012; Fan et al., 2019; Heidari et al., 2011; Zhai et al., 2016). In the case of kernel‐related traits, MTA for KW (*IWA6659*) was found to have a pleiotropic relationship with other kernel and spike traits including TKW and SL (Supplemental Table S4), and multi‐trait QTL have also been previously reported for grain yield, TKW, KW, NSPS, SL, and NKPS in wheat (Chen et al., 2016; Patil et al., 2013; Prashant et al., 2012; Wu et al., 2015). Pleiotropic effects may also partially be explained by correlations between agronomic traits (Chen et al., 2016; Kumar et al., 2016; Liu et al., 2017b), as observed in our study (Figure 2).

### 4.4 Additive effect of favorable alleles for wheat yield enhancement

Integrating multiple favorable loci into the same genetic background can significantly optimize plant traits (Fan et al., 2019; Li et al., 2021; You et al., 2021), which could be an effective strategy for modern wheat improvement. In our study, favorable alleles showed significant additive effects on the phenotype of four spike‐related traits (Figure 4). The lines carrying a combination of favorable alleles showed significantly greater trait performance than other lines without the alleles, indicating the importance of combining favorable alleles to improve the performance of yield‐contributing traits in wheat. Our study showed that the germplasm with three or more favorable alleles of identified QTL significantly improved the NSPS count compared with germplasm with only one favorable allele (Figure 4). Several of the desirable and undesirable lines, based on the highest and lowest phenotypic value for spike‐related traits, were compared to examine the cumulative allele effect on the individual germplasm phenotypes (Table 3). The line with the highest NSPS in our HWWAMP was MT9982 (19.87) followed by OK05108 (19.23), which had all four favorable alleles from four stable MTAs, whereas lines with only one favorable allele, such as TAM400 (12.80), TAM109 (13.27), and HV906‐865 (13.27), were among the worst (Table 3). Similarly, an additive effect of favorable alleles was observed for other traits (SL, SD, and NKPS) as well, such as a line with the highest mean SL in all the environments was OK1067274 (9.57 cm), which had all the three favorable alleles, whereas line MT85200 (6.49 cm), with the shortest SL, had only one favorable allele (Table 3). In our study, we also observed that many released varieties and advanced breeding lines from various breeding programs have a better combination of favorable alleles from stable MTAs detected for multiple spike‐related traits. For instance, one advanced line OK05108 was among the best for NSPS and SL, with all the seven favorable alleles from all stable MTAs detected for these two traits (Table 3).

**TABLE 3:** List of undesirable and desirable lines carrying low and high number of favorable alleles, respectively, for spike‐related traits

Trait	Entry name	No. of favorable alleles	Trait mean	Year of release	Origin^a^	Trait	Entry name	No. of favorable alleles	Trait mean	Year of release	Origin
NSPS	CUTTER	1	13.33	2002	KS	SD	SAGE	0	1.73	1973	KS
	TAM400	1	12.8	2001	TX		CO04025	0	1.78	–	CO
	TAMW‐101	1	13.53	1971	TX		LARNED	0	1.79	1976	KS
	TAM109	1	13.27	1991	TX		EAGLE	0	1.79	1970	KS
	HV906‐865	1	13.27	–	KS		NORKAN	0	1.83	1986	KS
	TX86A6880	4	18.43	–	TX		RAWHIDE	3	2.32	1990	NE
	MT9904	2	18.53	–	MT		ROSE	3	2.37	1979	SD
	MT9513	3	18.9	–	MT		MT9513	3	2.39	–	MT
	OK05108	4	19.23	–	OK		COSSACK	3	2.46	1998	KS
	MT9982	4	19.87	–	MT		MT9904	3	2.53	–	MT
SL	MT85200	1	6.49	–	MT	NKPS	TANDEM	1	26.45	1997	SD
	HOMESTEAD	1	6.69	1973	NE		HOMESTEAD	1	27.55	1973	NE
	HV906‐865	1	6.72	–	KS		G1878	1	28.08	–	KS
	OVERLEY	0	6.91	2004	KS		TRIUMPH64	1	28.6	1964	OK
	TRIUMPH64	2	6.95	1964	OK		AGATE	1	28.92	1979	NE
	OK05108	3	9.34	–	OK		RONL	2	49.65	2007	KS
	NEOSHO	3	9.42	2006	KS		TX06V7266	2	50.45	–	TX
	GUYMON	3	9.49	2005	OK		SMOKYHILL	1	51.17	2006	KS
	HAIL	3	9.51	1982	CO		TX05V7269	2	51.23	–	TX
	OK1067274	3	9.57	–	OK		HV9W06‐504	2	54.33	–	KS

*Note*. NSPS, number of spikelets per spike; SL, spike length; SD, spikelet density; NKPS, number of kernels per spike.

^a^ Origin represents the breeding program

In addition, there are many released cultivars from both before (‘Hail’, ‘Rose’, and ‘Cossack’) and in the early 21st century (‘Neosho’ ‘RonL’, and ‘SmokyHill’) that showed good phenotypic performance and were found to have a good combination of favorable alleles for multiple spike‐related traits (Table 3; Supplemental Table S1). Moreover, we observed that released cultivars and advanced breeding lines from recent decades have accumulated favorable alleles for various traits and this germplasm exhibited higher trait mean values than most of the cultivars released prior 1980s (Table 3). These findings suggest that continuous efforts by wheat breeders have resulted in the development of lines with a higher number of favorable alleles over time resulting in increased trait means. Such breeding lines and cultivars with a higher number of favorable alleles for multiple traits could serve as a valuable resource for the development of new high‐yielding varieties in the hard winter wheat breeding programs.

### 4.5 Spatial distribution of favorable alleles of stable MTAs

**FIGURE 5:**
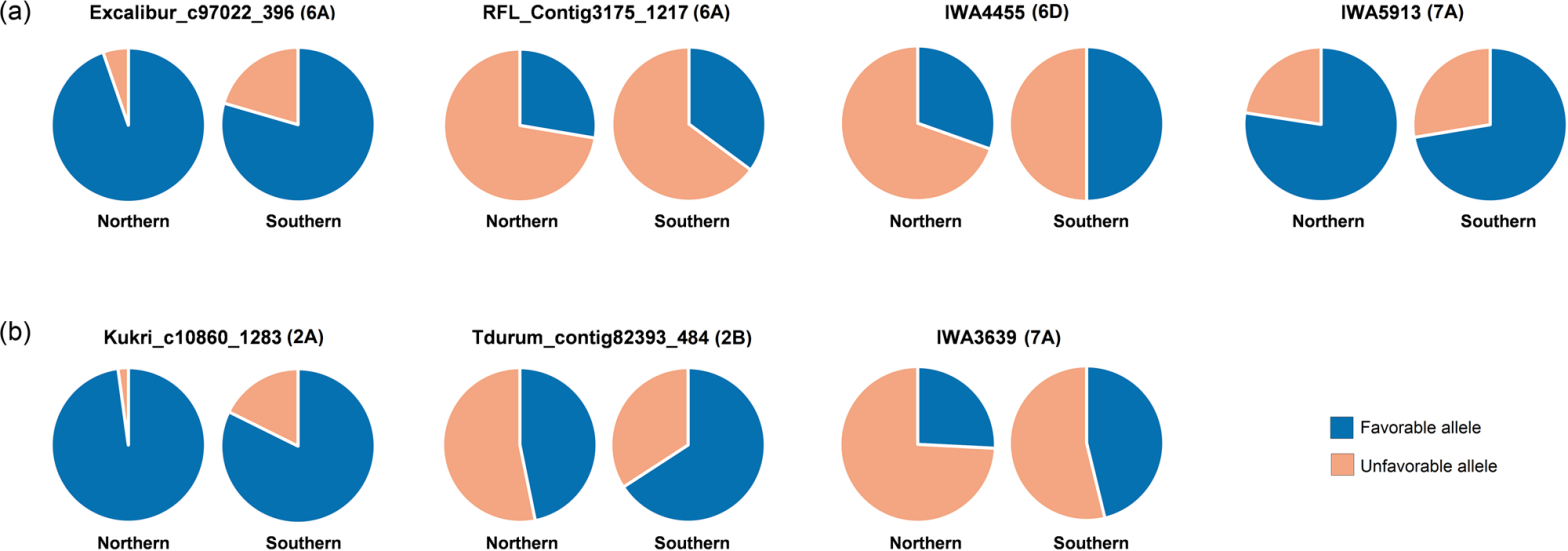
Pie charts showing the frequency distribution of favorable alleles of stable marker–trait associations (MTAs) in the germplasm from two broad wheat growing regions, Northern and Southern Great Plains for the two spike‐related traits: (a) number of spikelets per spike (NSPS, count) and (b) spike length (SL, cm). The ID of the associated SNP is given on the top of the pie charts with respective chromosome numbers in parenthesis

The spatial distribution of favorable alleles of stable MTAs was investigated by grouping 297 HWWAMP accessions based on their region of origin. We grouped the accessions into two broad groups: Northern and Southern Great Plains. The Northern group comprised accessions originating from the northern states including Montana, Nebraska, North Dakota, and South Dakota, whereas the Southern group included accessions from Colorado, Kansas, Oklahoma, and Texas. Out of the four stable genomic regions identified for NSPS, the MTA represented by *Excalibur_c97022_396* was observed to be fixed in germplasm from the Northern region and also exhibited high frequency in germplasm from the Southern region. This genomic region has been previously found to be associated with NSPS in multiple environments (Sun et al., 2017), suggesting a presence of stable QTL for this trait that might be effective in a broad range of environments. Apart from this, favorable allele for another NSPS‐associated MTA corresponding to the location of *WAPO1* was found to be accumulated in the majority of accessions from Northern and Southern regions. However, there is still an opportunity to use *WAPO1* in the Great Plains region, as about 25% of germplasm did not have the favorable allele (Figure 5). In addition to these two MTAs for NSPS, another genomic region represented by *IWA4456* was observed to be better distributed in germplasm from the Southern region, suggesting its role in adaptation to the Southern region.

Out of three stable MTAs for SL, the region represented by *Kukri_c10860_1283* was observed to be almost fixed in the Northern germplasm along with a high frequency of the favorable allele in the Southern germplasm, suggesting the importance of this genomic region in the Great Plains. In contrast, MTAs represented by *Tdurum_contig82393_484* and *IWA3639* were observed to be more frequent in germplasm from the Southern region (Figure 5).

### 4.6 Putative candidate genes for several important MTAs

Fourteen putative candidate genes were identified in the regions surrounding five MTAs (−log_10_ *P* ≥ 4.0) detected for different spike‐related traits (NSPS, SL, and SD) based on their potential involvement and higher expression in the shoot, spike, and kernel development at vegetative and reproductive stages of wheat (Table 2). Gene *TraesCS6A01G068900* was identified in the vicinity of an MTA for NSPS on chromosome 6A that is annotated to encode for the protein peptidyl‐prolyl *cis*‐*trans* isomerase (PPIase), which has a significant role in the deposition of storage proteins in wheat (Dutta et al., 2011). It was also found that PPIase activity in the wheat was regulated by the developmental stages and was also cultivar dependent (Dutta et al., 2011). Similarly, *TraesCS6A01G069500* encodes for cold shock protein (CSP), a family known for its role in cold acclimation, gene expression regulation, and developmental processes such as flower and seed development, among others (Behl et al., 2020). A field experiment conducted by Yu et al. (2017) found that the cold shock protein SeCspA significantly improved TKW and grain yield in wheat.

Several putative candidate genes were identified for SL encoding for proteins such as NADPH‐cytochrome P450 reductase, glycoprotein membrane glycosylphosphatidylinositol‐anchored, and others (Table 2), which might play a significant role in multiple processes of plant growth and development (Pandian et al., 2020). For instance, *TaMs1* (glycosylphosphatidylinositol‐anchored lipid transfer protein) is a wheat fertility gene, was found to express in pollen development and encodes a GPI‐LTP targeted to the plasma membrane (Kouidri et al., 2018). Protein ubiquitination is a major posttranslational modification that occurs in eukaryotes and regulates diverse biological processes such as lysine ubiquitination in common wheat, which mediates diverse cellular processes including carbohydrate metabolism, signal transduction, and photosynthesis (Zhang et al., 2017). Therefore, these putative genes may provide importance insights for understanding the underlying genetic mechanism of spike development in common wheat.

In conclusion, the current study showed that the HWWAMP is a valuable source for exploiting genetic variation for various yield‐contributing traits. The negative relationships between spike‐ and kernel‐related traits indicate that the loci controlling these traits are typically in an inverse relationship, leading to a moderate physiological trade‐off between primary grain yield components. Genome‐wide association studies effectively identified both stable and environment‐specific QTL for various spike‐ and kernel‐related traits. The SNP markers linked to these stable QTL identified in this study could be useful for marker‐assisted breeding in the hard winter wheat breeding programs and could also be incorporated into multivariate genomic selection models for the enhancement of yield‐related traits in wheat.

## AUTHOR CONTRIBUTIONS

Jyotirmoy Halder: Conceptualization, Data curation, Formal analysis, Investigation, Methodology, Software, Validation, Visualization, Writing – original draft, Writing – review & editing. Harsimardeep Gill: Data curation, Formal analysis, Investigation, Software, Visualization, Writing – review & editing. Jinfeng Zhang: Data curation, Investigation, Writing – review & editing. Rami Altameemi: Investigation. Eric Olson: Writing – review & editing. Brent Turnipseed: Writing – review & editing. Sunish Sehgal: Conceptualization, Funding acquisition, Methodology, Project administration, Resources, Supervision, Validation, Writing – original draft, Writing – review & editing.

## CONFLICT OF INTEREST

The authors declare that the research was conducted in the absence of any commercial or financial relationships that could be construed as a potential conflict of interest.

## Supporting information

Supplemental Table S1. Adjusted phenotypic means (BLUE) for eight different spike‐ and kernel‐related traits in hard winter wheat association mapping panel (HWWAMP).
Supplemental Table S2. SNP distribution across the three wheat sub‐genomes used for GWAS in the hard winter wheat association mapping panel (HWWAMP).
Supplemental Table S3. Mean comparison based on least significant difference (LSD) among four subpopulations (P1, P2, P3, and P4) for eight spike and kernel traits.
Supplemental Table S4. Marker‐trait associations identified in the combined environment (CEnv) by using trait BLUEs from across environment analysis.
Supplemental Figure S1. Pearson's linear correlation matrix among three different environments (E1, E2, and E3) and their best linear unbiased estimates (BLUE) designated as CEnv for spike‐related traits. Values inside the small square boxes represent the correlation coefficient. a) number of spikelets per spike (NSPS); b) spike length (SL); c) spikelet density (SD); d) number of kernels per spike (NKPS).
Supplemental Figure S2. Pearson's linear correlation matrix among three different environments (E1, E2, and E3) and their best linear unbiased estimates (BLUE) designated as CEnv for kernel‐related traits. Values inside the small square boxes represent the correlation coefficient. a) kernel width (KW); b) kernel length (KL); c) kernel area (KA); d) thousand kernel weight (TKW).
Supplemental Figure S3. Manhattan plots and quantile‐quantile (Q‐Q) plots of genome‐wide association studies using BLUEs from combined environments (CEnv) for a) number of spikelets per spike (NSPS); b) spike length (SL); c) spike density (SD); d) thousand kernel weights (TKW); e) number of kernels per spike (NKPS); f) kernel width (KW); and g) kernel area (KA). Y‐axis: ‐log10P and X‐axis: wheat chromosomes. The horizontal lines represent the threshold of ‐log10P ≥ 3 to report the marker‐trait associations.

## Data Availability

The genotyping dataset analyzed during the current study is available online (https://wheat.triticeaetoolbox.org/breeders/trial/2772). The data generated during this study are included in the Supplemental information file.
