# Supplementary material for: Genome‐wide association analysis of spike and kernel traits in the U.S. hard winter wheat
Source: Plant Genome. 2023 Jan 13;16(1):e20300. doi: 10.1002/tpg2.20300 (PMC12806900; doi:10.1002/tpg2.20300)
Supplement: Supplementary file 1 [file TPG2-16-e20300-s001.pdf]

Supplementary Information

Genome-wide association analysis of spike and kernel traits in the US hard winter wheat

Jyotirmoy Halder[1], Harsimardeep S. Gill[1], Jinfeng Zhang[1], Rami AlTameemi[1], Eric Olson[2], Brent Turnipseed[1], and Sunish K. Sehgal[1*]

[1] Department of Agronomy, Horticulture & Plant Science, South Dakota State University, Brookings, SD 57007

[2] Department of Plant, Soil and Microbial Sciences, Michigan State University, East Lansing, MI 48824

Supplementary Tables

Supplementary Table S1. Adjusted phenotypic means (BLUE) for eight different spike- and kernel-related traits in hard winter wheat association mapping panel (HWWAMP).

Sl. no.	Entry name	NSPS	SL	SD	NKPS	KW	KL	KA	TKW
1	2145	15.3	7.7	2.0	34.7	2.7	6.0	12.5	28.5
2	2180	16.0	8.6	1.9	39.9	2.7	6.2	12.8	27.6
3	2174-05	16.8	7.8	2.2	34.8	2.9	5.7	12.6	30.6
4	ABOVE	15.5	8.0	2.0	43.5	2.8	6.0	13.1	28.6
5	AGATE	14.8	8.3	1.8	28.9	3.0	6.8	15.2	36.4
6	AKRON	16.2	8.7	1.9	36.9	2.9	5.9	13.1	32.1
7	ALICE	16.1	8.6	1.9	42.5	2.8	6.0	13.1	29.2
8	ALLIANCE	16.3	8.2	2.0	41.1	2.8	6.3	13.6	30.6
9	ANTELOPE	15.9	8.3	1.9	38.4	2.8	5.9	12.3	29.0
10	ANTON	16.6	8.1	2.1	38.0	2.9	6.1	13.5	32.3
11	ARAPAHOE	15.2	8.5	1.8	34.7	2.7	6.4	13.3	30.3
12	ARLIN	14.8	8.4	1.8	37.1	2.9	6.1	13.5	32.8
13	AVALANCHE	15.4	8.2	1.9	41.7	2.9	6.2	13.7	30.1
14	BAKERS_WHITE	16.1	8.1	2.0	40.6	2.8	6.3	13.5	31.2
15	BENNETT	14.5	8.0	1.8	34.0	3.0	6.5	14.6	34.7
16	BIG_SKY	16.6	8.2	2.0	44.9	2.8	5.9	12.4	27.8
17	BILL_BROWN	15.4	8.3	1.9	41.1	2.8	6.2	13.5	29.2
18	BILLINGS	14.5	8.8	1.6	42.0	2.9	6.7	14.9	36.3
19	BISON	15.2	8.0	1.9	32.6	2.8	6.4	13.9	34.2
20	BOND_CL	15.0	8.8	1.7	44.5	2.8	6.0	13.1	28.2
21	BRONZE	15.7	8.3	1.9	29.3	2.7	6.1	12.5	28.7
22	BUCKSKIN	17.0	7.7	2.2	36.6	2.9	6.0	13.4	32.1
23	BURCHETT	16.3	8.1	2.0	38.0	2.7	6.0	12.4	29.2
24	BYRD	15.5	9.0	1.7	43.0	2.9	6.1	13.5	32.9
25	CAMELOT	16.7	8.6	1.9	40.4	2.9	6.4	14.0	34.3
26	CAPROCK	12.7	7.0	1.8	35.5	2.8	6.0	13.1	28.3
27	CARSON	16.9	7.7	2.2	42.8	2.8	5.9	12.6	28.9
28	CENTERFIELD	15.2	7.7	2.0	38.4	2.8	6.1	13.1	29.9
29	CENTURA	16.6	7.2	2.3	38.0	2.8	6.0	12.9	29.1
30	CENTURK78	15.7	7.2	2.2	31.4	2.6	5.9	11.9	26.7
31	CENTURY	15.7	8.2	1.9	41.4	2.7	6.0	12.5	28.4
32	CHENEY	15.4	8.3	1.9	32.5	2.7	6.1	12.7	28.4
33	CHEYENNE	16.4	7.5	2.2	31.3	2.7	6.1	12.7	30.0
34	CHISHOLM	14.3	7.9	1.8	34.3	2.9	6.4	13.9	34.7
35	CO03064	16.9	9.1	1.9	46.6	2.8	6.1	13.2	29.6

36	CO03W043	14.7	8.2	1.8	38.6	2.9	6.3	13.9	32.5
37	CO03W054	16.6	9.3	1.8	44.8	2.9	6.5	14.4	31.8
38	CO04025	15.2	8.5	1.8	43.6	2.9	6.0	13.6	30.2
39	CO04393	16.1	9.2	1.8	40.2	2.9	6.2	13.9	32.7
40	CO04499	13.8	7.8	1.8	42.8	2.9	6.2	13.9	32.0
41	CO04W320	16.5	9.2	1.8	46.6	2.9	6.1	13.8	31.7
42	CO050337-2	16.1	7.9	2.1	43.8	2.9	6.2	13.6	31.6
43	CO07W245	16.2	8.2	2.0	42.9	3.0	6.1	14.1	33.9
44	CO940610	15.1	8.4	1.8	38.3	3.0	6.4	14.5	35.6
45	COLT	15.3	7.2	2.1	33.6	2.7	6.2	12.6	31.0
46	COMANCHE	15.3	7.8	2.0	35.3	2.8	6.2	13.3	30.8
47	COSSACK	18.3	7.4	2.5	44.6	2.9	6.1	13.3	32.7
48	COUGAR	15.4	8.4	1.8	36.6	2.9	6.2	13.5	33.9
49	CREST	18.3	7.9	2.3	36.5	2.7	5.8	11.8	25.6
50	CRIMSON	17.9	7.8	2.3	34.1	2.6	5.7	11.3	27.0
51	CULVER	15.0	7.6	2.0	33.5	2.8	6.4	13.4	29.9
52	CUSTER	15.6	8.8	1.8	38.2	2.8	6.2	13.3	31.6
53	CUTTER	13.3	7.5	1.8	43.8	2.8	6.2	13.5	29.1
54	DANBY	17.2	8.5	2.0	44.3	2.8	6.1	13.4	31.3
55	DARRELL	14.9	8.2	1.8	35.9	2.8	6.4	13.4	30.3
56	DAWN	14.9	7.9	1.9	33.4	2.8	6.0	12.7	29.7
57	DECADE	15.1	8.1	1.9	44.1	2.8	6.1	12.8	28.8
58	DELIVER	17.8	9.0	2.0	40.4	2.9	6.4	14.1	31.6
59	DENALI	17.0	8.6	2.0	47.1	2.8	6.3	13.7	31.2
60	DODGE	14.8	8.0	1.8	31.7	2.7	6.0	12.5	28.9
61	DUKE	16.6	7.6	2.2	45.2	2.7	5.9	12.3	29.1
62	DUMAS	17.0	8.3	2.1	42.2	2.9	5.5	12.1	28.1
63	DUSTER	16.8	8.4	2.0	40.8	2.9	6.2	13.8	31.8
64	E2041	15.9	8.4	1.9	41.8	2.9	6.2	13.8	30.3
65	EAGLE	14.2	7.9	1.8	29.9	2.8	6.3	13.3	30.1
66	ENDURANCE	16.4	8.7	1.9	34.6	2.9	6.4	14.1	32.2
67	ENHANCER	15.5	8.0	1.9	44.8	2.7	6.2	12.9	27.4
68	EXPEDITION	13.9	7.3	1.9	34.1	2.8	6.3	13.5	32.4
69	FULLER	14.6	7.9	1.9	37.8	2.8	6.0	12.8	30.7
70	G1878	15.4	7.7	2.0	28.1	3.0	6.3	14.6	38.6
71	GAGE	15.9	8.3	1.9	32.5	2.8	6.1	13.0	31.0
72	GALLAGHER	18.3	8.4	2.2	44.7	2.8	6.4	13.8	31.7
73	GARRISON	14.2	7.5	1.9	41.1	2.9	5.9	13.1	29.4
74	GENOU	18.3	8.7	2.1	40.6	2.7	6.1	12.5	27.7
75	GENT	14.4	7.5	1.9	33.9	2.8	6.2	13.6	31.7
76	GOODSTREAK	14.1	7.6	1.9	38.8	2.9	6.3	13.7	33.8

77	GUYMON	17.2	9.5	1.8	43.9	2.8	6.5	14.0	29.7
78	HAIL	17.5	9.5	1.8	37.4	2.8	6.6	13.8	30.1
79	HALLAM	14.8	7.6	1.9	34.5	2.9	6.1	13.6	31.3
80	HALT	13.3	7.7	1.7	37.3	2.9	5.9	12.9	31.2
81	HARDING	16.7	8.1	2.1	38.4	2.8	6.3	13.3	31.7
82	HARRY	17.1	8.0	2.1	48.4	2.9	6.3	14.1	32.0
83	HATCHER	14.7	8.1	1.8	37.9	2.8	6.2	13.8	32.8
84	HEYNE	15.9	7.9	2.0	38.9	2.8	6.0	13.0	30.6
85	HG-9	18.0	9.0	2.0	35.9	3.0	6.4	14.2	34.2
86	HOMESTEAD	13.8	6.7	2.1	27.6	2.7	5.9	12.2	28.0
87	HONDO	15.2	7.6	2.0	34.2	2.9	6.3	14.1	35.0
88	HUME	17.3	8.0	2.2	36.7	2.7	5.8	11.8	26.6
89	HV906-865	13.3	6.7	2.0	34.7	2.8	5.7	12.3	27.6
90	HV9W03-1379R	16.1	7.1	2.3	47.2	2.7	5.7	11.7	25.4
91	HV9W03-1551WP	14.4	8.1	1.8	36.1	2.8	6.4	13.7	28.9
92	HV9W03-1596R	16.6	8.7	1.9	41.7	2.9	6.0	13.2	31.5
93	HV9W05-1280R	15.4	8.6	1.8	38.1	3.0	6.2	14.2	36.5
94	HV9W06-504	16.5	7.3	2.3	54.3	2.8	5.6	12.2	25.9
95	INFINITY_CL	15.8	7.4	2.1	40.4	2.8	5.9	12.7	29.4
96	INTRADA	14.7	7.8	1.9	31.9	2.8	6.1	13.0	31.4
97	JAGALENE	16.3	8.1	2.0	45.0	2.8	5.9	12.6	27.3
98	JAGGER	14.8	8.1	1.8	41.7	2.7	6.0	12.2	26.7
99	JERRY	16.6	7.5	2.2	34.6	2.8	6.6	14.4	35.0
100	JUDEE	17.2	8.5	2.0	36.2	2.7	5.9	12.0	27.1
101	JUDITH	17.2	8.5	2.0	41.7	2.8	6.7	14.6	30.2
102	JULES	16.4	8.2	2.0	43.4	2.8	6.1	13.1	28.0
103	KARL_92	15.2	8.1	1.9	34.4	2.8	6.3	13.5	32.0
104	KAW61	17.8	8.3	2.1	29.0	2.8	6.0	12.8	33.3
105	KEOTA	15.8	8.6	1.9	41.6	2.9	6.3	14.2	33.4
106	KHARKOF	16.7	8.9	1.9	35.3	2.8	5.9	13.0	32.4
107	KIOWA	14.9	7.8	1.9	29.7	2.9	6.3	13.7	33.0
108	KIRWIN	15.6	7.9	2.0	32.5	2.9	6.3	13.8	33.9
109	KS00F5-20-3	14.4	8.1	1.8	42.9	3.0	6.3	14.2	34.4
110	LAKIN	13.9	8.0	1.7	37.8	2.8	6.2	13.5	29.5
111	LAMAR	16.6	7.5	2.2	38.5	2.8	6.5	14.0	33.1
112	LANCER	15.7	7.2	2.2	30.9	2.7	5.9	12.1	29.2
113	LARNED	14.5	8.1	1.8	32.9	2.8	6.4	13.8	32.2
114	LINDON	15.3	8.2	1.9	41.1	2.7	5.9	12.5	29.4
115	LONGHORN	14.7	8.4	1.8	35.5	2.9	5.8	13.0	30.8
116	MACE	17.1	8.2	2.1	39.8	2.7	6.1	12.7	26.7
117	MCGILL	14.5	8.3	1.7	42.7	2.8	6.1	12.8	29.0

118	MILLENNIUM	15.2	7.0	2.2	34.6	2.9	5.9	12.6	30.1
119	MIT	13.5	7.8	1.7	37.8	2.7	5.9	12.6	29.0
120	MT0495	17.3	8.9	1.9	41.8	2.7	6.0	12.3	26.6
121	MT06103	17.1	8.1	2.1	40.4	2.9	6.2	13.3	32.8
122	MT85200	15.0	6.5	2.3	32.6	2.7	5.8	12.3	26.9
123	MT9513	18.9	7.9	2.4	42.3	2.8	5.9	12.5	25.7
124	MT9904	18.5	7.3	2.5	36.6	2.8	6.0	12.7	29.5
125	MT9982	19.9	8.8	2.3	43.1	2.8	6.1	13.2	29.8
126	MTS0531	16.0	8.5	1.9	38.7	2.8	6.4	13.5	26.4
127	NE02558	16.8	8.6	2.0	40.4	2.8	6.3	13.3	29.2
128	NE04490	15.8	7.8	2.0	42.9	2.8	6.1	13.2	29.9
129	NE05430	14.1	7.3	1.9	39.8	2.7	5.8	12.3	27.0
130	NE05496	17.3	8.4	2.1	41.7	2.8	6.0	13.0	29.6
131	NE05548	16.6	8.0	2.1	43.0	2.9	6.7	14.6	35.4
132	NE06545	15.1	8.2	1.8	36.0	2.9	6.0	13.5	31.0
133	NE06607	13.6	7.9	1.7	38.6	2.9	6.6	14.3	31.9
134	NE99495	16.4	8.8	1.9	38.8	2.8	6.4	13.6	30.0
135	NEKOTA	13.8	7.8	1.8	29.7	2.9	6.1	13.5	33.2
136	NELL	15.1	7.1	2.2	31.3	2.8	5.9	12.7	30.6
137	NEOSHO	17.2	9.4	1.8	47.7	2.9	5.8	12.8	30.0
138	NEWTON	16.1	8.7	1.9	39.9	2.8	6.1	13.0	29.1
139	NI06736	14.9	8.2	1.8	34.1	2.9	6.3	13.8	33.6
140	NI06737	14.8	7.8	1.9	43.3	2.8	6.0	12.6	28.0
141	NI07703	16.3	7.3	2.2	45.7	2.7	6.2	12.8	29.6
142	NI08707	15.3	8.2	1.9	46.2	2.9	6.6	14.7	34.3
143	NI08708	15.0	8.1	1.9	42.6	2.8	6.2	13.2	28.3
144	NIOBRARA	15.8	7.6	2.1	37.3	2.8	6.3	13.3	28.0
145	NORKAN	14.4	7.9	1.8	31.6	2.7	6.2	12.7	29.3
146	NORRIS	15.8	7.9	2.0	35.0	2.9	5.9	13.1	28.2
147	NUFRONTIER	17.6	8.0	2.2	49.3	2.7	6.0	12.2	25.3
148	NUHORIZON	16.0	8.1	2.0	38.9	2.8	5.8	12.4	28.8
149	NUPLAINS	15.8	7.9	2.0	37.1	2.7	5.9	12.2	28.1
150	NUSKY	16.8	7.5	2.3	40.9	2.7	5.9	12.3	27.0
151	NW03666	16.0	8.5	1.9	41.0	2.8	6.3	13.7	31.7
152	OGALLALA	15.0	7.5	2.0	36.6	2.7	5.6	11.6	27.0
153	OK_BULLET	18.3	8.9	2.0	42.8	3.0	6.4	14.6	33.8
154	OK_RISING	15.7	8.3	1.9	39.2	2.9	5.9	13.4	32.7
155	OK02405	17.0	8.1	2.1	43.3	2.9	5.8	12.9	31.1
156	OK04111	16.3	8.7	1.9	37.7	2.9	6.0	13.3	33.2
157	OK04415	16.8	7.3	2.3	35.9	2.9	6.0	13.3	31.4
158	OK04505	15.4	7.9	2.0	45.7	2.9	6.2	13.7	31.9

159	OK04507	17.1	8.3	2.1	40.0	2.9	6.1	13.5	32.8
160	OK04525	15.2	7.3	2.1	33.7	2.8	5.6	11.9	27.0
161	OK05108	19.2	9.3	2.1	37.3	2.9	6.2	13.9	32.0
162	OK05122	14.6	7.4	2.0	41.1	2.9	6.0	13.4	32.0
163	OK05134	15.9	8.6	1.9	41.9	3.1	6.3	15.0	36.8
164	OK05204	15.3	8.4	1.8	38.9	3.1	6.1	14.5	36.0
165	OK05303	15.5	8.3	1.9	34.5	3.0	6.1	14.1	34.9
166	OK05312	15.6	8.0	2.0	40.9	3.0	6.0	13.9	34.3
167	OK05511	17.4	8.4	2.1	42.8	3.0	5.8	13.2	32.8
168	OK05526	13.9	7.1	2.0	38.3	3.1	6.7	15.7	39.7
169	OK05711W	15.5	8.0	2.0	39.7	2.8	5.7	12.4	29.2
170	OK05723W	17.2	8.5	2.0	47.8	3.0	6.4	14.6	34.2
171	OK05830	16.3	8.2	2.0	40.1	2.9	5.6	12.4	29.4
172	OK06114	16.3	8.3	2.0	44.7	2.9	6.0	13.3	30.7
173	OK06210	15.0	8.3	1.8	38.4	3.0	6.2	14.1	34.3
174	OK06318	15.5	8.0	1.9	36.7	2.8	5.8	12.5	29.0
175	OK06319	15.7	8.4	1.9	38.9	2.9	5.9	13.4	33.1
176	OK06336	14.0	8.2	1.7	41.1	2.9	6.5	14.5	34.9
177	OK07231	15.6	7.8	2.0	42.3	2.8	6.2	13.2	31.0
178	OK07S117	16.3	8.6	1.9	39.3	2.9	6.3	13.8	29.9
179	OK08328	17.5	7.6	2.3	42.1	2.9	6.0	13.2	29.9
180	OK09634	13.6	7.1	1.9	41.0	2.8	6.1	12.8	28.8
181	OK101	15.2	8.7	1.8	36.5	2.8	6.2	12.9	29.4
182	OK10119	16.3	8.7	1.9	39.6	2.7	6.0	12.7	29.2
183	OK102	15.5	7.4	2.1	33.8	2.8	5.9	12.6	29.8
184	OK1067071	16.9	8.3	2.0	42.1	2.8	6.0	12.8	30.2
185	OK1067274	16.4	9.6	1.7	44.6	2.9	6.2	14.0	31.9
186	OK1068002	15.6	8.1	1.9	44.7	2.9	6.1	13.4	30.6
187	OK1068009	16.7	7.7	2.2	38.2	3.0	6.0	14.0	34.8
188	OK1068026	16.2	8.5	1.9	46.4	3.1	5.9	13.7	32.7
189	OK1068112	17.1	8.6	2.0	40.3	3.0	6.4	14.6	36.0
190	OK1070267	17.1	8.6	2.0	47.7	2.9	6.1	13.4	28.9
191	OK1070275	16.2	8.2	2.0	43.2	2.9	6.0	13.1	28.0
192	ONAGA	14.4	7.1	2.0	34.7	2.9	5.5	12.4	28.3
193	OVERLAND	14.8	7.0	2.1	36.3	2.8	6.2	13.2	30.0
194	OVERLEY	13.6	6.9	2.0	38.3	3.0	6.1	13.7	35.6
195	PARKER	16.5	7.4	2.2	34.0	2.8	6.0	12.7	30.6
196	PARKER76	15.7	7.6	2.1	34.0	2.8	6.1	12.8	30.4
197	PETE	14.9	9.3	1.6	34.6	2.9	5.8	13.3	31.1
198	PLATTE	16.3	8.7	1.9	49.0	2.8	5.8	12.6	27.3
199	POSTROCK	14.9	7.9	1.9	41.4	2.8	5.7	12.3	28.7

200	PRAIRIE_RED	13.7	7.5	1.8	38.7	2.9	6.2	13.8	31.2
201	PRONGHORN	16.0	7.4	2.2	38.7	2.9	6.4	14.1	34.0
202	PROWERS	17.0	7.9	2.2	34.9	2.9	6.5	14.4	36.7
203	RAWHIDE	16.1	7.0	2.3	42.3	2.8	6.1	12.8	27.2
204	REDLAND	16.0	7.6	2.1	34.5	2.8	6.0	12.7	29.4
205	RIPPER	14.5	8.8	1.7	36.1	2.9	6.5	14.3	31.6
206	RITA	16.4	8.0	2.1	39.1	2.7	5.9	12.1	26.7
207	ROBIDOUX	16.5	8.8	1.9	41.0	2.9	5.9	13.5	33.2
208	RONL	16.7	8.3	2.0	49.7	2.9	5.9	13.0	29.0
209	ROSE	17.8	7.5	2.4	39.9	2.7	5.9	11.9	28.0
210	ROSEBUD	18.2	8.0	2.3	35.9	2.8	6.0	12.8	31.5
211	SAGE	15.1	8.8	1.7	35.3	2.8	6.4	13.6	31.0
212	SANDY	15.9	7.8	2.1	37.3	2.7	5.9	12.4	28.3
213	SANTA_FE	13.7	7.7	1.8	41.7	2.9	6.1	13.3	30.9
214	SCOUT66	14.5	8.7	1.7	36.4	2.9	6.4	14.2	36.0
215	SD00111-9	15.2	8.3	1.8	37.3	2.9	6.8	15.0	35.9
216	SD01058	18.1	8.7	2.1	39.3	2.7	6.0	12.7	29.3
217	SD01237	14.1	7.9	1.8	37.5	2.9	6.3	14.1	34.1
218	SD05118	16.8	8.3	2.0	44.1	2.8	6.3	13.3	30.4
219	SD05210	16.8	8.4	2.0	35.0	2.8	6.1	13.0	30.8
220	SD05W018	15.6	7.4	2.1	43.3	2.8	6.2	13.0	28.7
221	SETTLER_CL	15.5	8.5	1.8	38.9	2.8	6.2	13.6	31.8
222	SHAWNEE	17.9	8.6	2.3	41.7	2.9	6.2	13.5	31.7
223	SHOCKER	14.2	7.5	1.9	43.4	2.9	5.9	13.1	30.8
224	SIOUXLAND	16.0	8.3	1.9	37.4	2.9	6.3	13.5	32.8
225	SMOKYHILL	16.5	7.7	2.2	51.2	2.7	5.9	12.3	27.5
226	SPARTAN	14.5	7.7	1.9	40.0	2.8	6.4	13.8	31.9
227	STANTON	15.0	7.6	2.0	38.4	2.9	6.2	13.5	30.9
228	STURDY	12.8	7.5	1.7	40.1	2.9	5.9	13.2	30.4
229	STURDY_2K	14.3	7.6	1.9	36.3	2.9	6.2	13.6	32.8
230	TAM105	14.1	8.0	1.8	31.8	2.8	6.0	13.2	29.8
231	TAM107	13.7	8.0	1.7	43.2	3.0	6.5	14.7	34.1
232	TAM107-R7	13.3	7.5	1.8	34.6	2.9	6.2	14.0	32.5
233	TAM109	13.3	7.8	1.7	30.0	2.9	6.5	14.2	33.8
234	TAM110	13.5	7.9	1.7	37.1	2.9	6.2	14.2	30.7
235	TAM111	15.8	7.7	2.1	43.9	2.8	5.9	12.7	29.9
236	TAM112	14.6	7.6	1.9	37.8	2.9	6.0	13.3	30.9
237	TAM200	14.2	7.3	1.9	37.8	2.7	5.9	12.4	29.6
238	TAM202	15.0	8.1	1.8	39.3	2.8	6.1	13.2	29.7
239	TAM203	13.7	7.0	2.0	41.3	2.8	6.2	13.5	28.2
240	TAM302	15.4	8.3	1.9	39.4	2.8	6.3	13.6	28.6

241	TAM303	13.7	7.6	1.8	39.1	3.0	6.1	14.2	32.8
242	TAM304	17.5	7.9	2.2	49.4	2.7	6.0	12.4	25.1
243	TAM400	12.8	7.6	1.7	36.2	2.8	5.9	12.5	29.1
244	TAM401	15.9	8.6	1.9	47.5	2.8	6.2	13.2	28.3
245	TAMW-101	13.5	7.4	1.8	30.1	2.9	6.6	14.5	36.6
246	TANDEM	14.4	7.5	1.9	26.5	2.9	6.2	14.0	36.1
247	TARKIO	15.0	7.1	2.1	39.2	2.9	6.2	13.7	30.4
248	TASCOSA	14.4	7.8	1.9	35.0	2.8	6.2	13.6	31.3
249	THUNDER_CL	15.8	9.1	1.7	43.6	2.9	6.2	13.6	28.6
250	THUNDERBOLT	14.6	7.6	1.9	37.7	2.9	5.8	12.8	31.5
251	TREGO	15.4	8.3	1.9	37.7	2.9	6.1	13.8	32.5
252	TRISON	15.7	7.9	2.0	32.2	3.0	6.2	14.3	38.5
253	TRIUMPH64	13.8	7.0	2.0	28.6	2.9	6.1	13.5	33.6
254	TURKEY_NEBSEL	17.3	8.7	2.0	36.6	2.9	6.3	13.6	33.2
255	TX00V1131	14.4	8.0	1.8	40.4	2.7	6.0	12.4	29.1
256	TX01A5936	16.1	8.7	1.9	46.9	2.9	6.3	13.9	33.0
257	TX01M5009-28	16.0	7.7	2.1	48.7	2.8	5.5	11.6	23.1
258	TX01V5134RC-3	13.9	8.0	1.8	44.1	2.9	6.1	13.6	32.2
259	TX02A0252	14.3	7.8	1.8	40.0	2.7	6.1	13.0	29.9
260	TX03A0148	16.4	9.3	1.8	45.3	2.9	6.5	14.5	32.2
261	TX03A0563	14.6	8.0	1.8	39.7	2.8	6.1	13.0	30.2
262	TX04A001246	15.1	8.6	1.8	41.2	2.9	6.1	13.7	34.3
263	TX04M410211	15.1	7.8	1.9	39.6	2.9	6.2	13.6	33.0
264	TX04V075080	14.5	8.2	1.8	40.6	2.9	6.7	14.6	32.4
265	TX05A001188	14.6	7.2	2.0	39.7	2.7	5.9	12.1	27.0
266	TX05A001822	15.7	8.2	1.9	45.3	2.9	5.9	13.3	32.1
267	TX05V7259	16.3	8.0	2.0	39.8	2.8	6.1	12.9	31.4
268	TX05V7269	17.2	8.3	2.1	51.2	2.7	6.0	12.5	26.4
269	TX06A001132	15.6	7.3	2.1	41.9	2.9	6.4	14.1	33.7
270	TX06A001263	16.3	7.8	2.1	43.0	2.8	5.9	12.9	28.9
271	TX06A001281	15.7	8.0	2.0	45.3	2.8	6.2	13.0	29.7
272	TX06A001386	16.2	8.8	1.8	41.5	2.8	5.9	12.8	30.7
273	TX06V7266	16.0	8.0	2.0	50.5	2.8	5.7	12.2	25.3
274	TX07A001279	16.2	7.4	2.2	46.7	2.7	5.6	11.8	26.2
275	TX07A001318	14.7	8.1	1.8	42.4	2.8	6.3	13.8	32.6
276	TX07A001420	15.9	8.6	1.9	42.5	2.9	5.8	12.7	30.3
277	TX86A5606	16.7	8.3	2.0	35.0	2.9	6.4	14.2	34.8
278	TX86A6880	18.4	8.8	2.1	42.4	2.9	6.3	13.7	32.7
279	TX86A8072	13.6	7.8	1.7	34.3	3.0	6.1	13.8	32.3
280	TX96D1073	14.2	7.6	1.9	35.1	2.9	5.8	12.9	30.4
281	TX99A0153-1	14.4	8.5	1.7	34.4	2.7	6.4	13.5	31.6

282	TX99U8618	15.3	8.0	1.9	37.0	2.7	5.8	12.1	29.1
283	VENANGO	16.4	8.3	2.0	42.2	2.8	6.1	13.2	32.0
284	VISTA	15.4	7.5	2.1	33.2	2.6	6.0	12.0	27.2
285	VONA	15.6	7.5	2.1	43.9	2.8	5.8	12.4	28.0
286	W04-417	13.6	7.3	1.9	45.2	2.8	5.8	12.4	26.9
287	WAHOO	16.3	7.7	2.1	36.9	2.8	5.8	12.2	27.7
288	WARRIOR	16.2	8.0	2.0	34.9	2.8	5.9	12.7	29.2
289	WB411W	15.9	8.0	2.0	42.9	2.9	6.3	13.5	31.1
290	WENDY	15.2	8.2	1.9	35.4	2.9	5.9	12.9	29.3
291	WESLEY	15.7	7.3	2.2	36.7	2.7	6.2	12.9	28.3
292	WICHITA	14.0	8.2	1.7	30.0	2.9	6.2	13.7	34.6
293	WINDSTAR	16.0	7.8	2.1	40.7	2.8	6.2	13.0	29.3
294	WINOKA	15.6	7.3	2.1	37.1	2.7	6.0	12.4	26.0
295	YELLOWSTONE	18.2	8.4	2.2	38.8	2.8	6.0	12.8	29.7
296	YUMA	15.3	7.9	1.9	37.9	2.8	6.2	13.4	31.6
297	YUMAR	15.7	8.0	2.0	45.2	2.8	6.0	13.1	28.0

NSPS, spikelet number per spike; SL, spike length; SD, spikelet density; NKPS, kernel number per spike; KW, kernel width; KA, kernel area; TKW, thousand kernel weight

Supplementary Table S2. SNP distribution across the three wheat sub-genomes used for GWAS in the hard winter wheat association mapping panel (HWWAMP).

Sub-genome	Chromosome	Number of SNPs	% SNPs
A	1	1,036	
	2	956	
	3	773	
	4	744	
	5	790	
	6	958	
	7	954	
Subtotal A	1-7	6,211	39.8
B	1	1,254	
	2	1,162	
	3	1,117	
	4	451	
	5	1,554	
	6	1,237	
	7	855	
Subtotal B	1-7	7,630	48.9
D	1	438	
	2	581	
	3	240	
	4	52	
	5	159	
	6	146	
	7	133	
Subtotal D	1-7	1,749	11.2
Total SNPs (A, B, and D)			100

Supplementary Table S3. Mean comparison based on least significant difference (LSD) among four subpopulations (P1, P2, P3, and P4) for eight spike and kernel traits.

Trait[€]	P1[$]	P2	P3	P4	*P*-value[#]
NSPS	16.05[a]	15.01[c]	15.31[bc]	15.58[b]	2.2e-05
SL	7.96[a]	8.06[a]	7.98[a]	8.13[a]	0.10
SD	2.03[a]	1.87[b]	1.92[b]	1.92[b]	3.46e-09
NKPS	37.86[c]	39.51[bc]	41.92[a]	39.7[b]	6.95e-05
KW	2.81[c]	2.9[a]	2.85[b]	2.83[b]	6.37e-08
KL	6.16[a]	6.16[a]	6.06[ab]	6.05[ab]	0.003
KA	13.21[b]	13.77[a]	13.2[b]	13.15[b]	0.0002
TKW	30.6[b]	32.16[a]	30.53[b]	30.53[b]	0.01

[€]NSPS, spikelet number per spike; SL, spike length; SD, spikelet density; NKPS, kernel number per spike; KW, kernel width; KL, kernel length; KA, kernel area; TKW, thousand kernel weight,

[$]Four sub-populations (designated as P1, P2, P3, and P4) based on the 'Structure' analysis

[#] Mean was compared based on LSD (least significant difference) with FDR (false discovery rate) corrected *P*-value at 0.05 level of significance. For each trait, values with the same letter indicates no significant difference in mean, while different letters indicate a significant difference ($P < 0.05$).

Supplementary Table S4. Marker-trait associations identified in the combined environment (CEnv) by using trait BLUEs from across environment analysis.

Trait[a]	SNP[$]	Chr	Allele	Pos	-Log$_{10}$(*P*)	effect	Environment*	Other trait(s)
NSPS	Excalibur_c35316_154	1A	T/C	2540768	4.3	0.20455	E2, CEnv	SD
	Excalibur_c11398_913	2A	C/T	118446604	3.4	-0.2223	CEnv	
	Kukri_c36783_91	2B	A/C	154985272	6.5	0.21808	E3, CEnv	
	Tdurum_contig42153_1190	2B	T/C	41198679	4.3	-0.2434	CEnv	
	BS00069271_51	4A	A/G	4620085	3.8	-0.2732	CEnv	
	BS00037357_51	4A	T/C	610493930	3.0	-0.2019	CEnv	
	BS00011235_51	5A	C/A	552515237	5.6	-0.3999	CEnv	
	IAAV4072	5A	C/A	42106388	4.3	-0.2106	E3, CEnv	
	Ex_c23426_1546	5B	A/G	40786594	3.6	0.27005	E2, CEnv	
	Excalibur_c97022_396	6A	C/T	37415157	4.0	-0.2661	E1, E3, CEnv	
	RFL_Contig3175_1217	6A	T/C	604877158	3.5	-0.1681	E2, E3, CEnv	
	IWA7896	6B	C/T	151130562	5.7	-0.3324	E3, CEnv	
	IWA4455	6D	A/G	462631946	3.8	0.06089	E1, E3, CEnv	
	IWA5913	7A	A/G	674276906	14.0	-0.5	E1, E2, E3, CEnv	SD
	GENE-4848_95	7B	G/T	739931213	4.6	0.20834	CEnv	
SL	TA006139-0953	1A	T/C	26959396	4.4	-0.107	E1, CEnv	
	RAC875_c60162_206	1A	T/C	545891404	3.5	-0.0836	CEnv	
	Kukri_c10860_1283	2A	G/A	87857405	4.7	0.16259	E1, E2, CEnv	
	Tdurum_contig82393_484	2B	C/A	730562664	8.4	0.14718	E1, E3, CEnv	
	IWA7916	2B	T/C	53464964	8.1	0.1535	E3, CEnv	
	IWA8127	3A	A/C	20004385	3.9	-0.0872	CEnv	
	Ra_c35_3184	3B	C/T	739984089	5.8	0.12681	E1, CEnv	
	Ex_c883_2618	4A	A/G	17259209	6.0	0.12594	E3, CEnv	
	Excalibur_rep_c68588_1196	4B	G/A	21377973	5.2	0.10603	CEnv	
	Kukri_c4210_480	4D	T/C	455252652	3.8	-0.1023	CEnv	
	CAP8_c1066_309	5A	A/G	488262170	3.3	0.15612	CEnv	

	JD_c63005_896	5B	T/C	513873396	3.3	-0.0947	E1, CEnv	
	IWA6116	6A	A/G	602708877	3.3	-0.1316	E2, CEnv	
	GENE-4204_311	6B	T/C	614437173	3.5	0.09673	CEnv	
	Tdurum_contig569_263	6B	T/C	704793345	3.0	0.08076	CEnv	
	IWA3639	7A	G/A	610934198	7.6	-0.1382	E1, E3, CEnv	
SD	Excalibur_c35316_154	1A	T/C	2540768	4.8	0.02645	CEnv	NSPS
	BobWhite_c14362_86	1B	T/C	653888017	3.9	0.05179	E3, CEnv	
	IAAV618	1D	T/C	486879128	4.9	-0.0346	CEnv	
	Kukri_rep_c104307_905	2A	A/G	32144831	3.6	-0.0244	CEnv	
	IWA2519	3A	C/T	371628644	3.3	-0.0258	E1, E2, CEnv	
	BobWhite_rep_c66057_98	4A	G/T	38369643	5.6	0.0307	CEnv	
	Kukri_c19760_2091	5B	A/G	177188132	4.1	-0.0194	E1, CEnv	
	RFL_Contig5037_560	6A	G/A	594748679	4.9	-0.0323	CEnv	
	TA015451-0472	6B	A/G	222005447	3.9	0.02838	E1, CEnv	
	IWA5913	7A	A/G	674276906	9.4	-0.0548	E1, E2, E3, CEnv	NSPS
	IWA1902	7D	A/G	530035575	4.1	-0.0411	E1, E2, E3, CEnv	
NKPS	IWA6805	1D	A/G	429698652	3.0	1.22743	CEnv	
	CAP8_c5108_139	2B	G/A	26567970	3.4	-2.3829	CEnv	
	BS00021959_51	2B	C/T	110818850	3.2	-1.8616	E1, E3, CEnv	
	Excalibur_c1921_1191	5B	G/A	427650909	4.0	1.38977	E1, E2, CEnv	
	IWA4329	5B	C/T	694520891	3.2	-1.3803	E2, CEnv	
KW	RAC875_c21411_162	1A	A/G	539964977	3.2	0.03634	CEnv	
	BS00044274_51	2A	T/G	47826702	3.3	0.02359	E2, E3, CEnv	KA
	IWA6659	4A	C/T	84934131	3.2	-0.0304	E1, CEnv	KA, TKW, SL
KA	IWA6485	3D	A/G	600261870	3.3	0.20109	CEnv	SL
	Kukri_c74409_199	4A	G/A	37773890	3.4	0.25033	E1, E3, CEnv	
TKW	Tdurum_contig46797_585	2A	T/C	44836524	3.3	0.725	E3, CEnv	

Chr, chromosome; Pos, physical position in base pair (based on IWGSC RefSeq).

*Environment 1(E1), Environment 2 (E2), Environment 3 (E3), across enviroment BLUEs (CEnv)

[a]NSPS, number of spikelets per spike; SL, spike length; SD, spikelet density; NKPS, number of kernels per spike; KW, kernel width; KA, kernel area; TKW, thousand kernel weight

Supplementary Figures

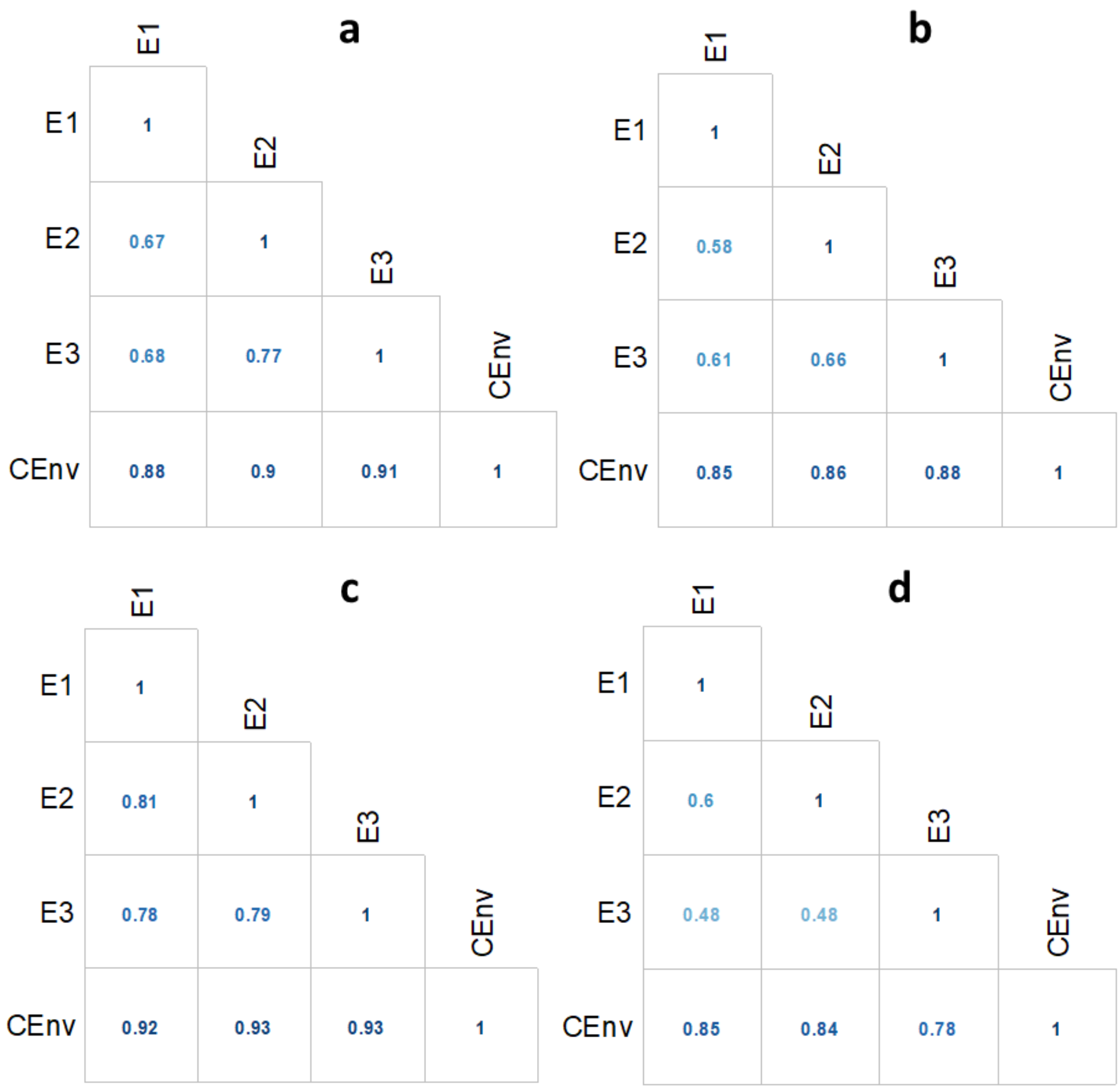

Supplementary Figure S1. Pearson's linear correlation matrix among three different environments (E1, E2, and E3) and their best linear unbiased estimates (BLUE) designated as CEnv for spike-related traits. Values inside the small square boxes represent the correlation coefficient. a) number of spikelets per spike (NSPS); b) spike length (SL); c) spikelet density (SD); d) number of kernels per spike (NKPS).

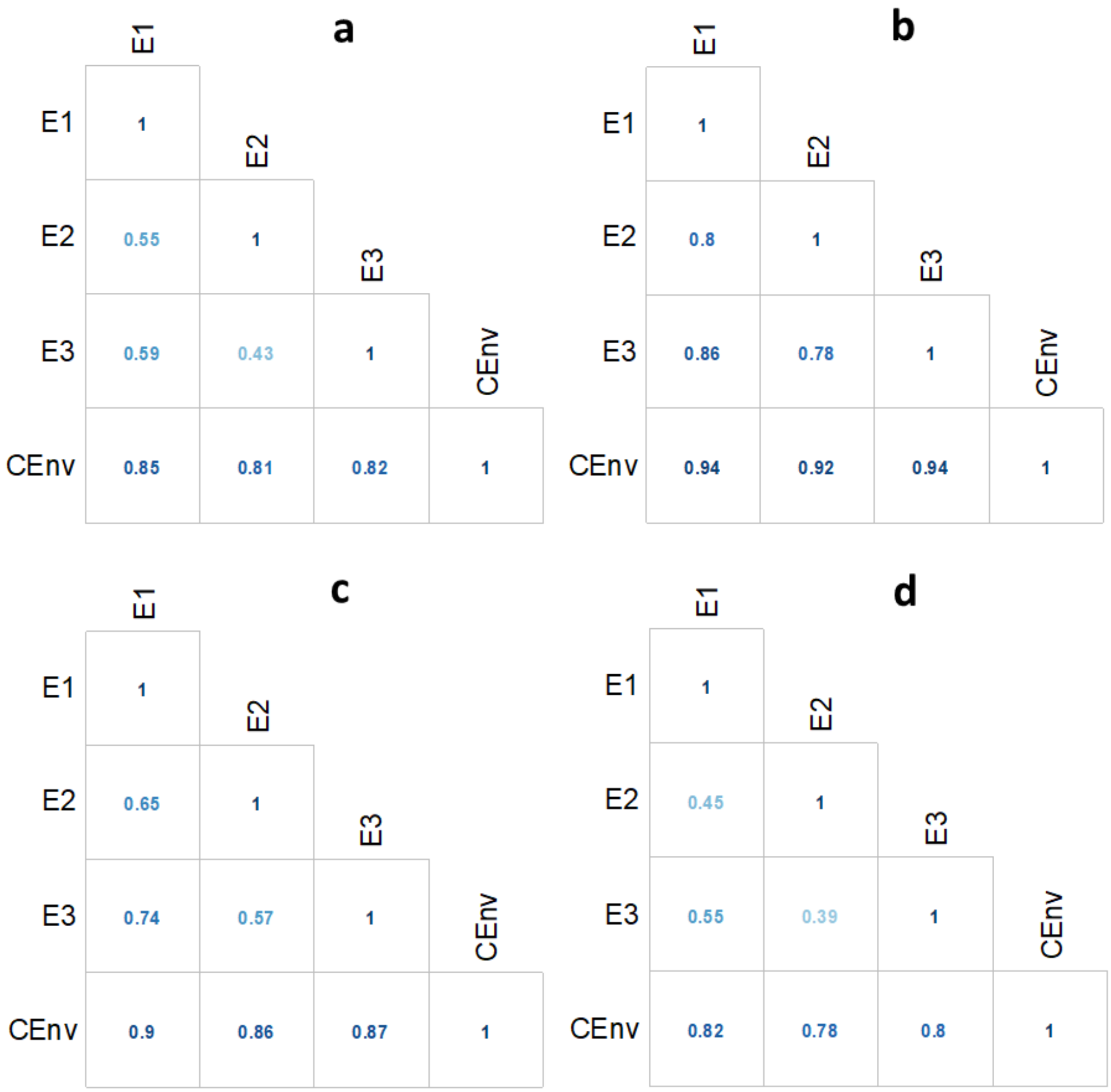

Supplementary Figure S2. Pearson's linear correlation matrix among three different environments (E1, E2, and E3) and their best linear unbiased estimates (BLUE) designated as CEnv for kernel-related traits. Values inside the small square boxes represent the correlation coefficient. a) kernel width (KW); b) kernel length (KL); c) kernel area (KA); d) thousand kernel weight (TKW).

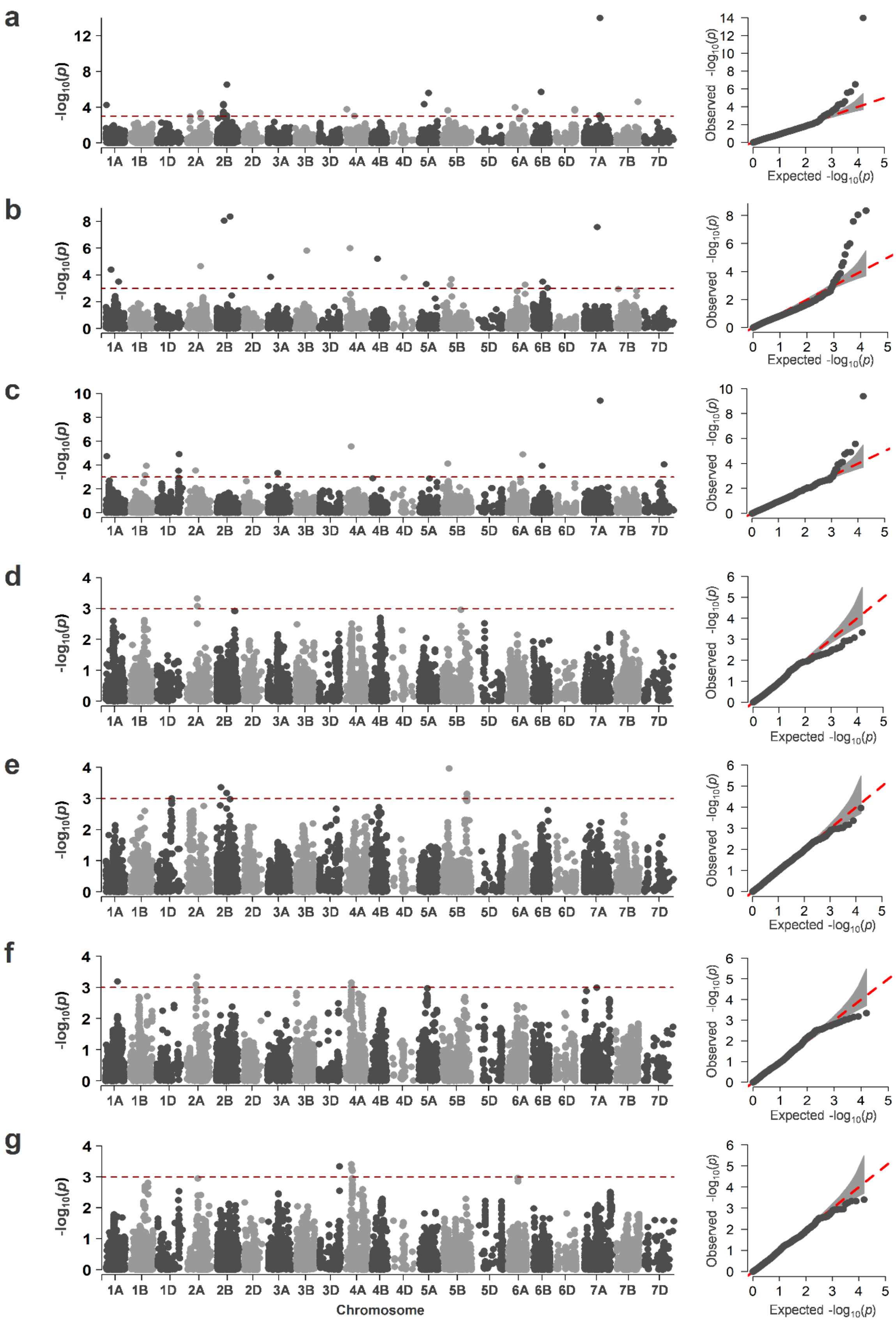

a
b
c
d
e
f
g
-log10(p)
Observed -log10(p)
Expected -log10(p)
1A 1B 1D 2A 2B 2D 3A 3B 3D 4A 4B 4D 5A 5B 5D 6A 6B 6D 7A 7B 7D
Chromosome

Supplementary Figure S3. Manhattan plots and quantile-quantile (Q-Q) plots of genome-wide association studies using BLUEs from combined environments (CEnv) for a) number of spikelets per spike (NSPS); b) spike length (SL); c) spike density (SD); d) thousand kernel weights (TKW); e) number of kernels per spike (NKPS); f) kernel width (KW); and g) kernel area (KA). Y-axis: -log10*P* and X-axis: wheat chromosomes. The horizontal lines represent the threshold of -log10*P* ≥ 3 to report the marker-trait associations.
